# High IL-1R8 expression in breast tumors promotes tumor growth and contributes to impaired antitumor immunity

**DOI:** 10.18632/oncotarget.17713

**Published:** 2017-05-09

**Authors:** Luis Felipe Campesato, Ana Paula M. Silva, Luna Cordeiro, Bruna R. Correa, Fabio C.P. Navarro, Rafael F. Zanin, Marina Marçola, Lilian T. Inoue, Mariana L. Duarte, Martina Molgora, Fabio Pasqualini, Matteo Massara, Pedro Galante, Romualdo Barroso-Sousa, Nadia Polentarutti, Federica Riva, Erico T. Costa, Alberto Mantovani, Cecilia Garlanda, Anamaria A. Camargo

**Affiliations:** ^1^ Ludwig Institute for Cancer Research, São Paulo, São Paulo, Brazil; ^2^ Molecular Oncology Center, Hospital Sírio-Libanês, São Paulo, São Paulo, Brazil; ^3^ Graduate Program in Biochemistry, Institute of Chemistry, University of São Paulo, São Paulo, Brazil; ^4^ Humanitas Clinical and Research Center, Rozzano, Italy; ^5^ Cellular and Molecular Immunology Laboratory, Pontifícia Universidade Católica do Rio Grande do Sul, Porto Alegre, Rio Grande do Sul, Brazil; ^6^ Institute of Biosciences, University of São Paulo, São Paulo, Brazil; ^7^ Department of Medical Oncology, Dana-Farber Cancer Institute, Boston, MA, USA; ^8^ Department of Veterinary Pathology, University of Milan, Milan, Italy; ^9^ Humanitas University, Rozzano, Italy

**Keywords:** breast cancer, SIGIRR/IL-1R8, Toll/IL-1 receptors, innate immune sensing, immune evasion

## Abstract

Tumors develop numerous strategies to fine-tune inflammation and avoid detection and eradication by the immune system. The identification of mechanisms leading to local immune dysregulation is critical to improve cancer therapy. We here demonstrate that Interleukin-1 receptor 8 (IL-1R8 - previously known as SIGIRR/TIR8), a negative regulator of Toll-Like and Interleukin-1 Receptor family signaling, is up-regulated during breast epithelial cell transformation and in primary breast tumors. IL-1R8 expression in transformed breast epithelial cells reduced IL-1-dependent NF-κB activation and production of pro-inflammatory cytokines, inhibited NK cell activation and favored M2-like macrophage polarization. In a murine breast cancer model (MMTV-neu), IL-1R8-deficiency reduced tumor growth and metastasis and was associated with increased mobilization and activation of immune cells, such as NK cells and CD8^+^ T cells. Finally, immune-gene signature analysis in clinical specimens revealed that high IL-1R8 expression is associated with impaired innate immune sensing and T-cell exclusion from the tumor microenvironment. Our results indicate that high IL-1R8 expression acts as a novel immunomodulatory mechanism leading to dysregulated immunity with important implications for breast cancer immunotherapy.

## INTRODUCTION

During tumorigenesis, cancer cells interact with the immune system and modify the tumor microenvironment (TME) to subvert the host immune response [1, 2]. In advanced tumors, the immune balance is tilted towards pro-tumor inflammation and local dysregulation of the innate and adaptive immune response [3]. Inflammatory signals derived from tumor cells, stromal cells and immune cells act as critical factors regulating qualitative and quantitatively pro-tumoral inflammation and antitumor immunity during the cancer-immunity cycle [3]. This complex crosstalk has been recognized as a hallmark of cancer [4], and understanding the molecular pathways involved in the induction of pro-tumoral inflammation and immune evasion could enable the development of new therapies and improve the efficacy of existing ones.

The activation of Interleukin-1 (IL-1) receptor family members (ILRs) and Toll-like receptors (TLRs) represents a critical early innate immune sensing event promoting immunosurveillance and antitumor immunity [5–7]. ILRs and TLRs activation ignites a signal transduction cascade with pro-inflammatory outcomes, including the activation of NF-κB and the secretion of pro-inflammatory cytokines such as Type I IFNs and TNFα, which are in turn necessary for natural killer (NK) and dendritic cell (DC) activation, together with CD8^+^ T cell priming against tumor antigens and trafficking into the TME [8–10]. Therefore, ILRs and TLRs activation plays an important role in inflammation, initiation and amplification of innate immunity and orientation of adaptive immunity [11, 12].

IL-1R8 is a member of the ILRs family. IL-1R8 negatively regulates signaling from IL-1R1, IL-18R, ST2 and a number of TLRs [13, 14] by acting as a decoy receptor for key components of the MyD88 signaling cascade, such as IRAK and TRAF6 and by interfering with ILRs dimerization through its Ig domain [13–15]. Recently, it has been demonstrated that, in humans, IL-1R8 can also bind to IL-37, an anti-inflammatory cytokine induced by TLRs and cytokines and a natural suppressor of innate inflammatory response [16, 17]. Curiously, no IL-37 homologue has been identified in mice wherein.

Irrespectively of its mode of action, IL-1R8 acts as a brake for pro-inflammatory signals, and its expression is essential for regulating the detrimental effects of innate and adaptive immune responses in pathologic conditions such as infections, sepsis, chronic inflammation and autoimmune disease [18–21]. In addition, IL-1R8-deficiency in mice results in more severe gut inflammation during dextran sulfate sodium colitis [22, 23], increased susceptibility to colitis-associated colorectal cancer [23–25], and more severe and earlier onset of monoclonal B cell expansions in a murine model of chronic lymphocytic leukemia (CLL) [26].

In this study, we show that IL-1R8 is up-regulated during breast epithelial cell transformation and in primary breast tumors. Using clinical samples and *in vitro* and *in vivo* experiments, we also demonstrate that high expression of IL-1R8 in breast tumors modulates the expression of inflammatory mediators in the TME, affecting the mobilization and activation of immune cells and fostering tumor growth and metastasis. Collectively, our findings indicate that expression of IL-1R8 represents a novel immunomodulatory mechanism leading to impaired innate immune sensing and antitumor immunity and provides new insights to cancer immunotherapy.

## RESULTS

### IL-1R8 is up-regulated in transformed breast epithelial cells and in primary breast tumors

**Figure 1:**
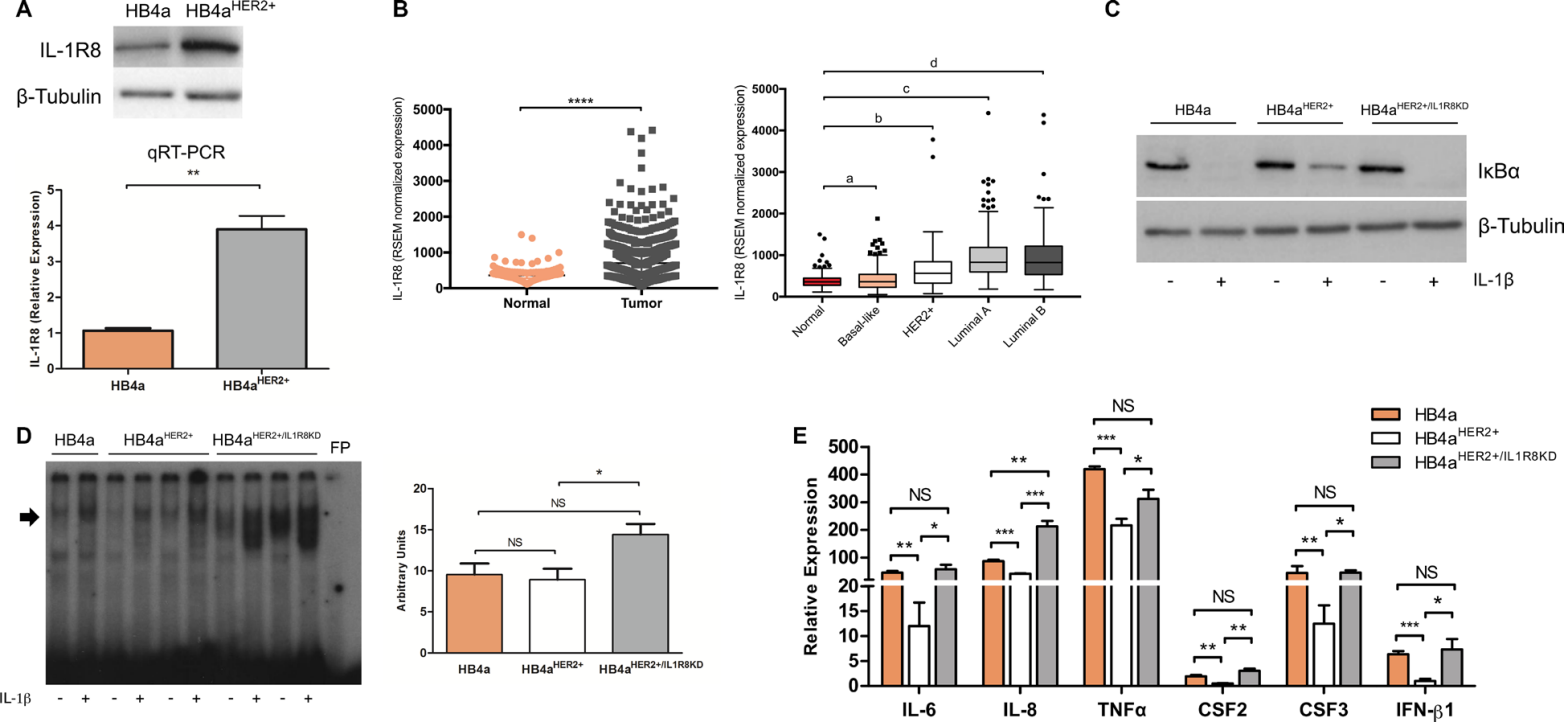
Up-regulation of IL-1R8 expression inhibits IL-1-dependent NF-κB activation and expression of pro-inflammatory cytokines in HER2-transformed breast cells (**A**) IL-1R8 protein expression by western-blot (upper part) and mRNA relative expression by qRT-PCR (lower part) in HB4a and HB4a^HER2+^ epithelial mammary cell lines. **P = 0.002, unpaired Student's *t*-test. (**B**) On the left, IL-1R8 normalized expression values in normal mammary tissue (*n* = 113) compared to primary breast tumors (*n* = 792); on the right, normal mammary tissue compared to Basal-like (*n* = 136), HER2+ (*n* = 65), Luminal A (*n* = 415) and Luminal B (*n* = 176) molecular breast cancer subtypes using RNA-seq data obtained from TCGA. a) *P* = 0.8, b) *P* = 1.1e^−08^, c) *P* = 2.2e^−16^, d) *P* = 2.2e^−16^, Wilcoxon rank-sum`s test. Data is shown as the group median value in RSEM normalized expression ± interquartile range. (**C**) Protein levels of IκB and β-Tubulin by Western-blot in HB4a, HB4a^HER2+^ and HB4a^HER2+/IL1R8KD^ cells stimulated or not with 5 ng/mL of IL-1β for 15 minutes (**D**) Electromobility shift assay (EMSA) for NF-κB of nuclear extracts of cells stimulated or not with IL-1β 5 ng/mL for 24 hours. Arrow indicates the position of NF-κB complex; FP: Free probe. Right panel: densitometry analysis of band intensity. (**E**) Cytokines expression of HB4a, HB4a^HER2+^ and HB4a^HER2+/IL1R8KD^ cells stimulated with IL-1β 5 ng/mL for 1 hour by qRT-PCR. Values represent expression relative to non-treated cells. Error bars indicate the variation between the means of three independent experiments. Unpaired Student's *t*-test *P < 0.05, **P < 0.01, ***P < 0.001, ***P < 0.0001, NS: not significant.

IL-1R8 was identified as an up-regulated gene in transformed breast epithelial cells by comparing gene expression profiles from a parental, non-transformed, conditionally immortalized human mammary luminal epithelial cell line (HB4a), and a HER2 overexpressing variant (HB4a-C5.2, designated HB4a^HER2+^ for the purpose of this work) [27]. Transcriptional changes associated with breast epithelial cell transformation were measured using Massively Parallel Signature Sequencing (MPSS) and IL-1R8 ranked among the top 50 differentially expressed genes (unpublished results). Reliable MPSS tags (5′GATCATAGGGACAGCGG3′) assigned to IL-1R8 were more frequently found in the HB4a^HER2+^ library than in the HB4a library (36 tpm vs. 4 tpm, *P* < 0.001), indicating that IL-1R8 gene expression is up-regulated in the transformed breast epithelial cells. IL-1R8 differential expression in the HB4a^HER2+^ variant was confirmed both at the mRNA and protein levels. A 4-fold induction of IL-1R8 mRNA and a 2-fold induction of IL-1R8 protein expression were observed in HB4a^HER2+^ cells when compared to HB4a (Figure 1A).

IL-1R8 up-regulation in primary breast tumors was confirmed by analyzing RNA-seq expression data obtained from The Cancer Genome Atlas (TCGA). We observed that IL-1R8 gene expression is significantly higher in primary breast tumors compared to normal breast tissue (median 701.1 vs. 358.8 RSEM normalized expression values, *P* < 0.0001, Figure 1B) and higher levels of IL-1R8 mRNA were observed across all molecular breast cancer subtypes, except in the basal-like breast cancer subtype (HER2^+^ subtype median 563.4 RSEM normalized expression values, *P* = 1.13e^−05^, Luminal A subtype median 830.2 RSEM normalized expression values, *P* < 2.2e-16, Luminal B median 823.9 normalized expression values, *P* < 2.2e-16 and basal-like subtype median 360.9 normalized expression values, *P* = 0.83) (Figure 1B).

Collectively, these results indicate that IL-1R8 is up-regulated during breast epithelial cell transformation and across all molecular breast cancer subtypes, except in the basal-like subtype.

### IL-1R8 up-regulation in transformed breast epithelial cells fine-tunes IL-1-dependent NF-κB activation and the expression of pro-inflammatory cytokines

IL-1R8 negatively regulates the innate inflammatory response by acting as a decoy receptor for TLRs and ILRs signaling. NF-κB activation and the production of pro-inflammatory cytokines are important endpoints of TLR and IL-1R family signaling [28]. Gene transfer experiments have shown that IL-1R8 up-regulation inhibits NF-κB activation and the production of pro-inflammatory cytokines in HeLa and hepG2 cells after exposure to IL-1 and TLR ligands [14]. Therefore, to address the role of IL-1R8 up-regulation in transformed breast epithelial cells, we treated HB4a and HB4a^HER2+^ cells with IL-1β and analyzed IκB expression levels and NF-κB activation by Western blot and EMSA, respectively. IκB is negative regulator of NF-κB and is phosphorylated by specific kinases in response to inflammatory signals. The phosphorylated IκB protein is then ubiquitinated and degraded leading to a decrease in IκB expression levels and to NF-κB activation. As expected, IκB expression decreases shortly after IL-1β treatment in both cell lines, but the decrease was less pronounced in HB4a^HER2+^ cells compared to HB4a (Figure 1C). To verify if the observed differences in IκB expression levels were due to IL-1R8 up-regulation in HB4a^HER2+^ cells, we generated HB4a^HER2+^ cell variants stably expressing IL-1R8 shRNAs (HB4a^HER2+/IL1R8KD^) (Supplementary Figure 1). No differences in the growth rate between HB4a^HER2+^ and HB4a^HER2+/IL1R8KD^ cells studied herein were observed and IL-1R8 knockdown remained stable during the course of this work (data not shown). After IL-1R8 knockdown, the differences in IκB expression levels observed between HB4a and HB4a^HER2+^ disappeared (Figure 1C). In addition, NF-κB activation was significantly enhanced in HB4a^HER2+/IL1R8KD^ cells compared to HB4a^HER2+^ cells (Figure 1D).

Gene-expression of different pro-inflammatory cytokines in HB4a and HB4a^HER2+^ cells was then analyzed after stimulation with IL-1β. Shortly after stimulation, a significant increase in IL-6, IL-8, CSF2, CSF3, IFN-β1 and TNFα expression was observed in both cell lines, although the induction of these inflammatory mediators was significantly lower in HB4a^HER2+^cells compared to HB4a cells. Noteworthy, IL-1R8 knockdown in HB4a^HER2+^ cells reverted the expression of these cytokines after IL-1β stimulation to the levels observed in parental HB4a cells (Figure 1E).

Taken together, these results indicate that IL-1R8 up-regulation in transformed breast epithelial cells attenuates IL-1-dependent NF-κB activation and the expression of pro-inflammatory cytokines.

### IL-1R8 deficiency in a transgenic mouse model of breast cancer delays tumor onset and reduces tumor burden and metastasis

**Figure 2:**
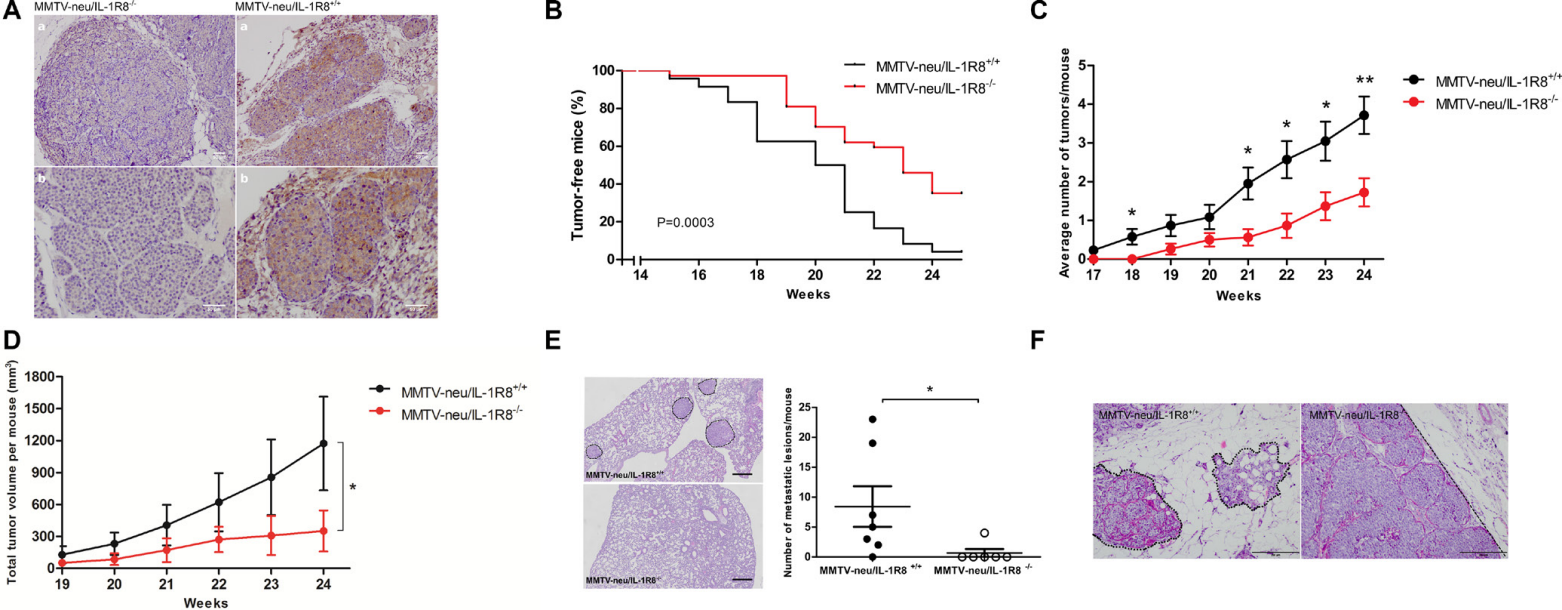
IL-1R8-deficiency delays tumor development and reduces tumor burden in a transgenic MMTV-neu animal model (**A**) IHC staining of IL-1R8 in mammary tumors from MMTV-neu/IL-1R8^+/+^ and MMTV-neu/IL-1R8^−/−^ mice. Scale bar: 50 μM. (**B**) Kaplan–Meier analysis of tumor-free survival of MMTV-neu/IL-1R8^+/+^ (*n* = 27) and MMTV-neu/IL-1R8^−/−^ (*n* = 37) mice. Log-rank test, *P* = 0.0003. (**C**) Average number of mammary tumors per mouse over time (weeks). *P < 0.05 and **P < 0.01, unpaired Student's *t*-test. (**D**) Average total tumor volume per mouse over time (weeks). *P = 0.024, paired Student's *t*-test. (**E**) Number of lung metastatic lesions per mouse at 30 weeks of age, Mann-Whitney test. Left panel, dotted line reveals regions of metastatic lesions. Scale bar: 50 μM. *P = 0.024. (**F**) Hematoxylin and eosin (HE) staining of mammary tumors from 24-weeks old mice revealing distinct growth and invasive features. MMTV-neu/IL-1R8^+/+^ infiltrative growth pattern (dotted line) and MMTV-neu/IL-1R8^−/−^ expansive growth pattern (dotted line). Scale bar: 200 μM.

To further investigate the role of IL-1R8 in breast tumor formation and progression, IL-1R8 knock-out mice (IL-1R8^−/−^) were crossed with a transgenic model of spontaneous breast tumorigenesis (MMTV-neu), to generate MMTV-neu/IL-1R8^−/−^ mice. The presence and absence of IL-1R8 expression in tumors from MMTV-neu/IL-1R8^+/+^ and MMTV-neu/IL-1R8^−/−^ mice, respectively, were confirmed by immunohistochemistry (IHC) and, in tumors from MMTV-neu/IL-1R8^+/+^ animals, IL-1R8 expression was predominantly detected in the tumor cells (Figure 2A).

Significant differences in tumor onset, incidence and burden were observed between the two groups of animals. As shown in Figure 2B, MMTV-neu/IL-1R8^−/−^ mice presented later tumor onset compared to MMTV-neu/IL-1R8^+/+^ mice. By week 24, 65% (24/37) of the animals in the MMTV-neu/IL-1R8^−/−^ group developed breast tumors compared to 95% (26/27) in the group of MMTV-neu/IL-1R8^+/+^ mice (*P* = 0.0003). In addition, at week 24, the mean number of mammary tumors per animal was significantly lower in the MMTV-neu/IL-1R8^−/−^ group as compared to the MMTV-neu/IL-1R8^+/+^ group (2 vs. 4 tumors/animal, *P* = 0.007, Figure 2C). Moreover, by week 24, tumors from MMTV-neu/IL-1R8^−/−^ mice were 3 times smaller than tumors from MMTV-neu/IL-1R8^+/+^ mice (351 vs. 1173 mm^3^, *P* = 0.02, Figure 2D). Finally, by week 30, the number of metastatic lesions in the lungs of MMTV-neu/IL-1R8^−/−^ mice was significantly lower than in MMTV-neu/IL-1R8^+/+^ animals (0.7 vs. 8.4 lesions/mouse, *P* = 0.03, Figure 2E).

Bone marrow chimeras were then used to assess the relative importance of IL-1R8 expression in the non-hematopoietic/tumor cells in the reduced tumor burden and growth observed in MMTV-neu/IL-1R8^−/−^mice. No significant differences in tumor onset, incidence and burden were observed when MMTV-neu/IL-1R8^−/−^mice were transplanted with IL-1R8^+/+^ bone-marrow cells, supporting an important role for IL-1R8 expression by non-hematopoietic/tumor cells during breast tumorigenesis (Supplementary Figure 2).

In addition to the differences observed in tumor growth and burden, tumors from MMTV-neu/IL-1R8^−/−^mice and MMTV-neu/IL-1R8^+/+^ mice displayed different histopathological characteristics. By week 24, tumors from MMTV-neu/IL-1R8^−/−^mice displayed fewer aggressive features, such as tissue necrosis and cellular atypia, although no difference was observed in the number of mitosis per field when compared to MMTV-neu/IL-1R8^+/+^ tumors (1.2 vs 2.4 mitosis/field, *P* = 0.22, Supplementary Table 1). Furthermore, MMTV-neu/IL-1R8^−/−^tumors exhibited an expansive growth pattern (100% vs 33%) while MMTV-neu/IL-1R8^+/+^ tumors were more infiltrative (66% vs 0%, *P* = 0.06, Supplementary Table 1 and Figure 2F).

These results reveal that IL-1R8 expression in breast tumor cells contributes to tumor formation, progression and metastatic dissemination.

### IL-1R8-deficiency in a transgenic mouse model of breast cancer promotes the mobilization and skews the activation of immune cells

Pro-inflammatory cytokines are critical factors regulating the mobilization and activity of immune cells in the TME [3]. Since IL-1R8 can negatively modulate the expression of inflammatory mediators in IL-1R-stimulated transformed breast epithelial cells *in vitro*, we sought to analyze its role on the immune composition *in vivo* using our transgenic mouse model of breast cancer.

**Figure 3:**
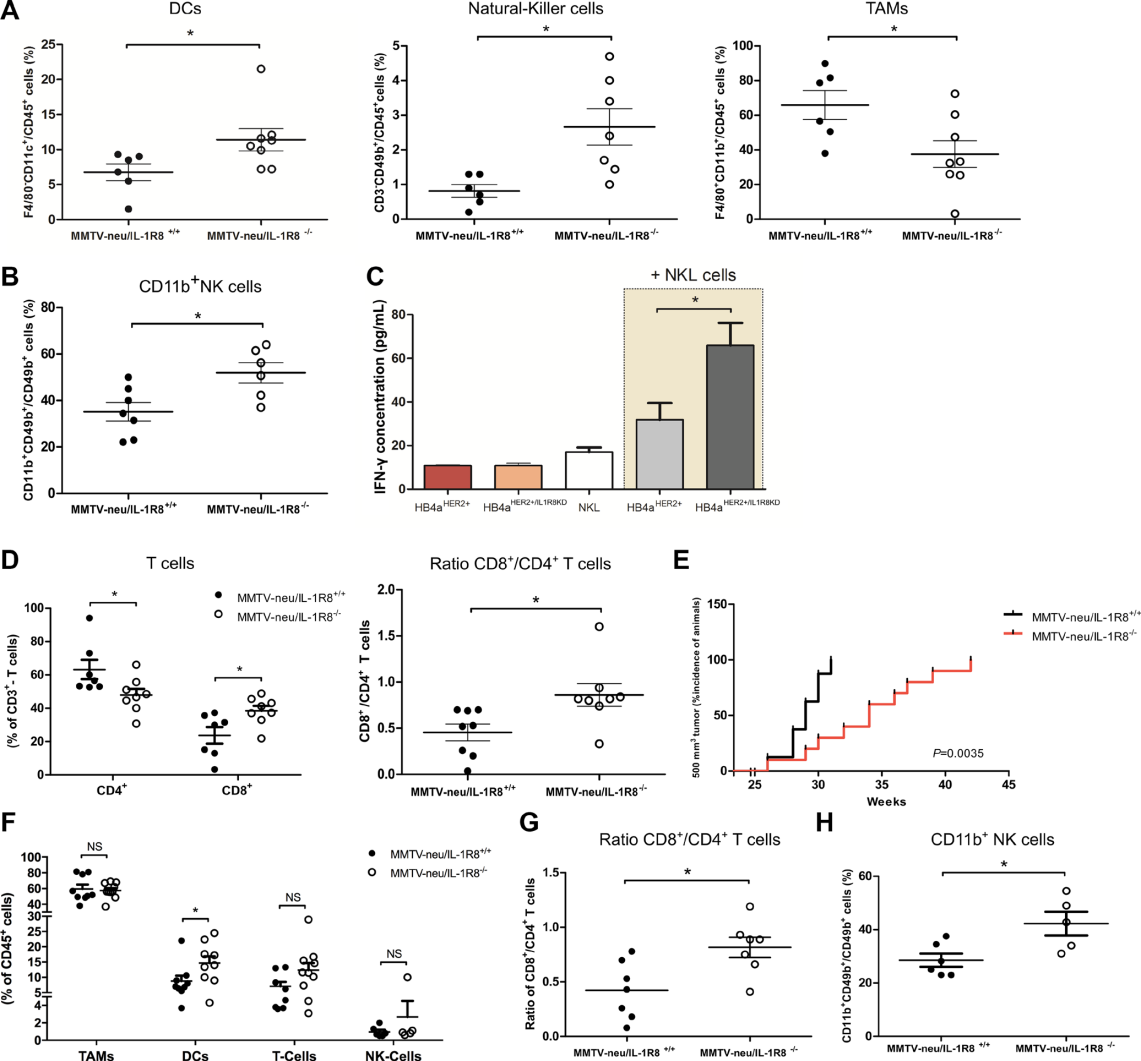
IL-1R8-deficiency promotes a protective tumor immune infiltrate in MMTV-neu mice Leukocyte infiltrate analysis by FACS (**A**) DCs (CD11b^+^F4/80^−^CD11c^+^), Natural-Killer cells (CD3^−^CD49b^+^), Macrophages (CD11b^+^F4/80^+^) and (**B**) Mature NK cell population (CD11b^+^CD49b^+^/Total NK cells). (**C**) IFN-γ levels determined by ELISA in the supernatants of NKL cells, HB4a^HER2+^ or HB4a^HER2+/IL1R8KD^ cells cultured separately or in co-culture with NKL cells (beige background). (**D**) FACS analysis of CD4^+^ and CD8^+^ T cells. Results are presented as % of CD3^+^ cells (left panel) or as ratio of CD8^+^ and CD4^+^ T cells (right panel). (**E**) Kaplan–Meier analysis of the incidence of tumors reaching 500 mm^3^ volume in MMTV-neu/IL-1R8^+/+^ (*n* = 8) and MMTV-neu/IL-1R8^−/−^ (*n* = 10) mice. *P* = 0.0035, Log-rank test. (**F**) FACS analysis of TAMs (CD11b^+^F4/80^+^), DCs (CD11b^+^F4/80^−^CD11c^+^), T-Cells (CD3^+^) and NK cells (CD3^−^CD49b^+^). Results are presented as % of CD45^+^ cells. (**G**) Ratio of CD8^+^/CD4^+^ T cells. (**H**) Mature NK cell population (CD11b^+^CD49b^+^/Total NK cells). Error bars indicate the variation between the means of three independent experiments. *P < 0.05, unpaired Student's *t*-test.

Although the total number of tumor-infiltrating leukocytes (CD45^+^ cells) from mice at 24-weeks of age was found to be lower in MMTV-neu/IL-1R8^−/−^ tumors (IRA 1.2 ± 0.2 vs. 2.4 ± 0.4, *P* = 0.02, Supplementary Figures 3A and 5), these tumors presented a significantly higher proportion of DCs (11.4 ± 1.6 vs. 6.8 ± 1.2 F4/80^−^CD11c^+^/CD45^+^ cells, *P* = 0.04) and NK cells (2.7 ± 0.5 vs. 0.8 ± 0.2 CD3^−^CD49b^+^/CD45^+^ cells, *P* = 0.01) and a lower proportion of Tumor Associated Macrophages (TAMs) (37.6 ± 7.7 vs. 65.9 ± 8.3 CD11b^+^F4/80^+^/CD45^+^ cells, *P* = 0.03) compared to MMTV-neu/IL-1R8^+/+^ tumors (Figure 3A and Supplementary Figures 3B, 5). A marginal difference in the percentage of immature macrophages (3.6 ± 0.8 vs. 9.4 ± 2.4 CD11b^+^F4/80^+^Ly6C^+^ cells/Macrophages, *P* = 0.052, Supplementary Figure 4A) was also observed between MMTV-neu/IL-1R8^−/−^ and MMTV-neu/IL-1R8^+/+^ tumors, but no significant differences in the proportion of infiltrating polymorphonuclear cells (PMN) (CD11b^+^Ly6G^+^/CD45^+^ cells, *P* = 0.9, Supplementary Figure 4B), B cells (CD19^+^/CD45^+^ cells, *P* = 0.6, Supplementary Figure 4C) or T cells (14.3 ± 3.9 vs. 18.7 ± 5.5 CD3^+^/CD45^+^ cells, *P* = 0.5, Supplementary Figure 4D) were observed between both types of tumors.

It is well known that the outcome of an immune response towards a tumor is largely determined by the type of immune response elicited and a tumor-directed immune response, involving NK cells and cytotoxic CD8^+^ T cells is known to protect against tumor development and progression [29]. NK cells constitutively express a lytic machinery capable of killing target cells independently of antigen presentation [30]. In addition to the higher proportion of NK cells observed in MMTV-neu/IL-1R8^−/−^ tumors, we found that these cells were also more mature (52.9 ± 4.4 vs. 35.2 ± 4 CD11b^+^ cells/NK cells, *P* = 0.02, Figure 3B). To further investigate whether IL-1R8 up-regulation in tumor cells could modulate NK cell activation, NKL cells were cultured in the presence of either HB4a^HER2+^ or HB4a^HER2+/IL1R8KD^ cells and IFN-γ levels in the culture supernatant were used as a surrogate marker of NK cell activation. Higher IFN-γ levels were detected in supernatants of NKL cells co-cultured with HB4a^HER2+/IL1R8KD^ as compared to HB4a^HER2+^ (65.9 ± 10.4 vs. 31.8 ± 7.7 pg/mL, *P* = 0.03) (Figure 3C), indicating that high IL-1R8 expression in tumors cells can potentially inhibit NK cell activation.

Besides NK cells, tumor infiltrating lymphocytes (TILs) also play and important role in tumor cell elimination and the presence of TILs in tumor biopsies, in particular of cytotoxic CD8^+^ T cells, is emerging as an independent positive prognostic factor in different solid tumors, including breast cancer [31]. Although we did not observe significant differences in the proportion of infiltrating T cells between MMTV-neu/IL-1R8^−/−^ and MMTV-neu/IL-1R8^+/+^ tumors Supplementary Figure 4D, MMTV-neu/IL-1R8^−/−^ tumors presented a significantly lower infiltrate of CD4^+^ T cells (47.9 ± 3.7 vs. 63.2 ± 5.1 CD4^+^/CD3^+^, *P* = 0.04) and higher infiltrate of CD8^+^ T cells (38.5 ± 3 vs. 23.7 ± 4.9 CD8^+^/CD3^+^, *P* = 0.02), with a higher CD8^+^/CD4^+^ T cell ratio (0.9 ± 0.1 vs. 0.4 ± 0.1, *P* = 0.02, Figure 3D), suggesting that IL-1R8 expression by tumors cells reduces the effective mobilization of CD8^+^ T cells into the TME.

We also analyzed tumors of equivalent sizes (tumor volume of 500 mm^3^), to evaluate whether the differences in the immune infiltrate could be due to different tumor sizes. As shown in Figure 3E, MMTV-neu/IL-1R8^−/−^ mice developed 500 mm3 tumors at later points compared to MMTV-neu/IL-1R8^+/+^ mice (Median of 34 vs. 29 weeks, *P* = 0.003). Notably, a higher percentage of DCs (14.7 ± 2.1 vs. 8.8 ± 1.8 F4/80^−^CD11c^+^/CD45^+^ cells, *P* = 0.04, Figure 3F) as well as a higher CD8^+^/CD4^+^ T cell ratio (0.8 ± 0.1 vs. 0.4 ± 0.1, *P* = 0.01, Figure 3G) was still observed in MMTV-neu/IL-1R8^−/−^ tumors. However, in contrast to tumors collected at 24-weeks, we did not observe significant differences in the percentage of infiltrating TAMs, T cells or NK cells between the tumors (Figure 3F) although a larger population of mature NK cells (42.3 ± 4.4 vs. 28.5 ± 2.5 CD11b^+^NK cell/total NK cells, *P* = 0.02, Figure 3H) was observed in MMTV-neu/IL-1R8^−/−^ tumors.

**Figure 4:**
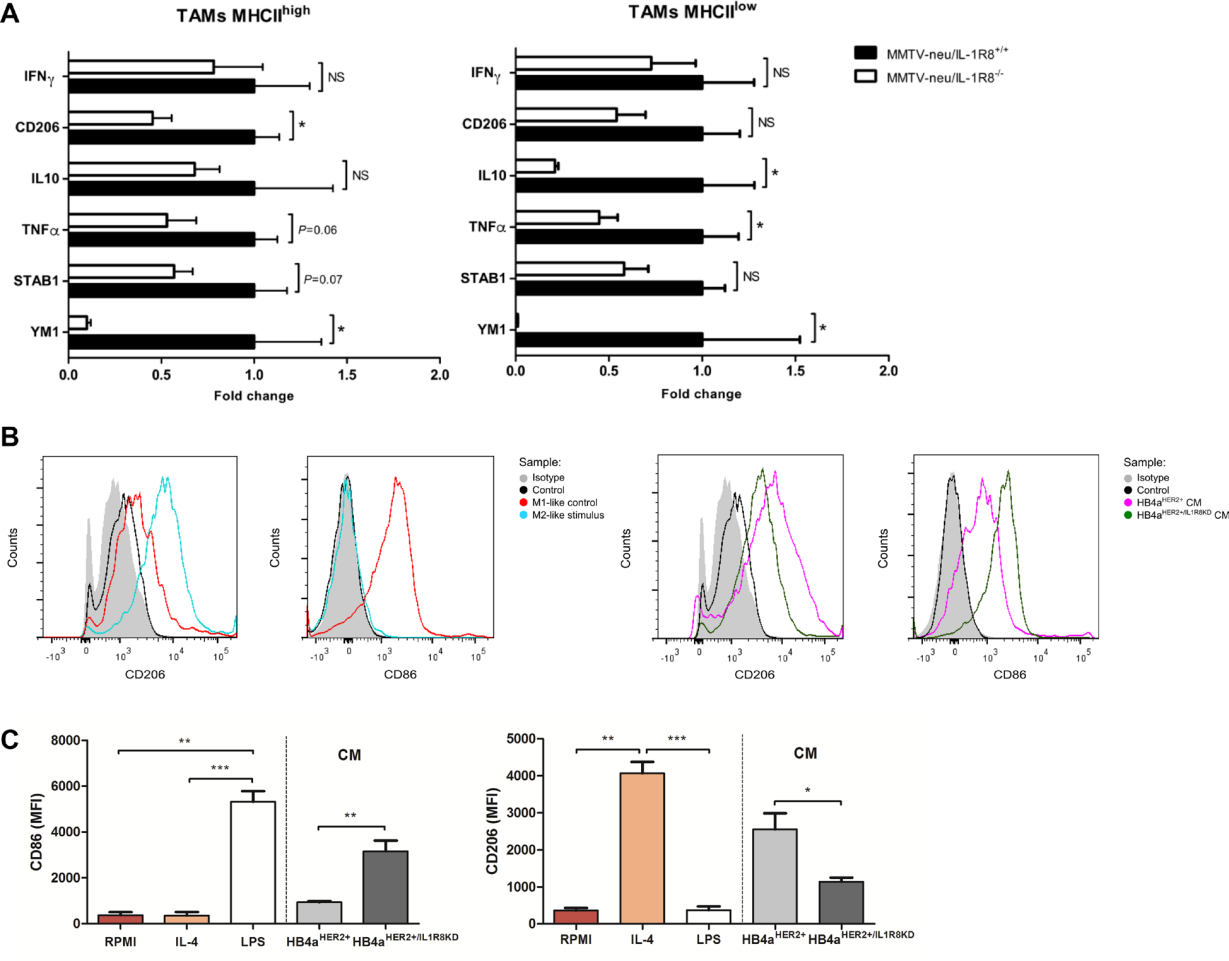
IL-1R8 expression modulates the polarization state of TAMs in mammary tumors (**A**) Analysis by qRT-PCR of selected M1 and M2-like markers of MHC^high^ and MHC^low^ tumor-associated macrophages (TAMs), infiltrating mammary tumors in MMTV-neu/IL-1R8^+/+^ (*n* = 4) and MMTV-neu/IL-1R8^−/−^ (*n* = 4) mice (mean ± SEM). Data were relative to 18S expression and normalized versus the mean of wild-type and expressed as mean ± SEM. (**B**) FACS histograms and (**C**) Mean Fluorescence Intensity (MFI) showing M1-like marker CD86 and M2-like marker CD206 expression in THP-1 macrophage-like cells after incubation with IL-4 30 ng/mL (M2-like polarization control), LPS 25 ng/mL (M1-like polarization control) or HB4a^HER2+^and HB4a^HER2+/IL1R8KD^ conditioned medium (CM) for 24 hours. (A–C) Error bars indicate the variation between the means of three independent experiments. *P < 0.05, **P < 0.01, ***P < 0.001, unpaired Student's *t*-test.

Since TAMs are known to generally present a pro-tumoral M2-like phenotype [32], we next characterized the phenotype of tumor-infiltrating myeloid cells from MMTV-neu/IL-1R8^−/−^ and MMTV-neu/IL-1R8^+/+^ tumors of the same size, looking at classic M1 and M2 polarization markers [33]. After purification of CD11b^+^/Ly6G^−^ cells, MHCII^high^ (CD11b^+^/Ly6C^−^/MHCII^high^) and MHCII^low^ (CD11b^+^/Ly6C^−^/MHCII^low^) TAMs were sorted to > 98% purity to analyze the expression of M1-like and M2-like markers as previously described (Supplementary Figure 6) [33]. As shown in Figure 4A, both MHCII^high^ and MHCII^low^ population of TAMs from MMTV-neu/IL-1R8^−/−^ tumors displayed lower levels of M2-like markers (CD206, Ym1, TNFα, STAB1, IL-10) compared to TAMs from MMTV-neu/IL-1R8^+/+^ tumors. To further investigate if IL-1R8 expression in tumor cells could induce M2-macrophage polarization, THP-1 macrophage-like cells [34] were cultured in the presence of conditioned medium from IL-1β-stimulated HB4a^HER2+^ and HB4a^HER2+/IL1R8KD^ cells. THP-1 macrophage-like cells cultured with conditioned medium from HB4a^HER2+^ expressed significantly higher levels of the mannose receptor M2-like marker CD206 (Mean Fluorescence Intensity (MFI) 2,551 ± 43 vs. 1,135 ± 11, *P* = 0.034) and lower levels of the T cell co-stimulatory M1-like marker CD86 (MFI 936 ± 38 vs. 3,157 ± 472, *P* = 0.009) as compared to HB4a^HER2+/IL1R8KD^ cells (Figure 4B and 4C), indicating that high IL-1R8 expression can skew macrophage polarization towards an M2-like phenotype.

We finally compared the intratumoral levels of different cytokines in homogenates from MMTV-neu/IL-1R8^−/−^ and MMTV-neu/IL-1R8^+/+^ tumors (Supplementary Figure 7). At 24-weeks, MMTV-neu/IL-1R8^−/−^ tumors presented lower intratumoral levels of IL-1β (273.2 ± 56 vs 667.6 ± 165 pg/mg, *P* = 0.02) and of pro-angiogenic vascular endothelial growth factor (VEGF) (152.5 ± 10.3 vs 279.7 ± 37.5 pg/mg, *P* = 0.004), but higher levels of IFN-γ (50.2 ± 8.6 vs 27.8 ± 4 pg/mg, *P* = 0.04). Accordingly, high intratumoral levels of IL-1β were recently shown to activate endothelial cells to produce VEGF and IL-1β inhibition stably reduced tumor growth by limiting inflammation and inducing the maturation of immature myeloid cells into M1 macrophages [35].

Collectively, our results suggest that IL-1R8 expression in breast tumors plays a critical role in the maintenance of a local pro-tumoral inflammatory microenvironment and prevents the development of protective immunity.

### IL-1R8 expression in clinical samples is associated with impaired mobilization of NK, DC and CD8^+^ T cells

The relative abundance of tumor-infiltrating leukocytes in clinical samples can be indirectly quantified by measuring intratumoral transcript levels of coordinately expressed immune cell-specific genes (immune metagenes) [36]. To determine the clinical relevance of our *in vivo* and *in vitro* findings, we analyzed RNA-seq expression data from 1102 primary breast tumors, including all four major molecular subtypes obtained from TCGA. Breast tumors were classified according to IL-1R8 expression levels irrespectively of their molecular subtype (see Materials and Methods) and were analyzed for the expression of immune metagenes.

In accordance with our findings, lower expression levels of T cell metagenes (CD28, CD3G, CD8A, CD8B, FYB, ICOS, LCP2, LTA) and of different chemokines associated with CD8^+^ T cell recruitment (CCL2, CCL3, CCL4, CCL5, CXCL9 and CXCL10) were observed among breast tumors expressing higher levels of IL-1R8 (IL-1R8 high tumors, Table 1). Furthermore, lower expression levels of T cell co-stimulatory markers (CD80, CD86), of DC metagenes and of components of the peptide-presenting machinery (HLA-DMA, HLA-DMB, HLA-DOA and HLA-DOB) were also observed in IL-1R8 high tumors (Supplementary Table 2). Finally, lower levels of several pro-inflammatory cytokines (IL1B, IL10, IL12, IL6, IL8, TNF), NK cell metagenes (NCR1, XCL1), cytolytic enzymes (GZMA, GZMB, PRF) and type I IFN-induced genes were also observed in IL-1R8 high breast tumors (Table 1 and Supplementary Table 2).

**Table 1:** Expression levels of immune-related genes in IL-1R8-high and IL-1R8-low primary breast tumors

		IL-1R8 High	IL-1R8 Low	adj-P
**T-Cell transcripts**	CD28	43.3	63.5	1.3E-08
	CD3G	16.2	31.0	1.6E-09
	CD8A	138.5	171.7	1.5E-02
	CD8B	35.1	49.6	7.4E-04
	FYB	314.8	484.6	7.0E-15
	ICOS	15.2	28.8	1.6E-09
	LCP2	290.8	397.3	5.2E-10
	LTA	9.0	12.0	3.9E-05
**CD8+ T-cell Chemokines**	CCL2	290.0	413.1	7.0E-08
	CCL3	85.4	102.2	1.7E-03
	CCL4	72.9	99.5	2.6E-08
	CCL5	441.4	577.3	1.9E-03
	CXCL9	475.2	977.6	2.1E-09
	CXCL10	314.5	571.5	9.1E-12
**IFN-induced genes**	EIF2AK2	438.5	624.1	1.8E-23
	GBP1	713.7	1258.3	1.8E-23
	IFI16	1402.9	2043.8	3.2E-21
	IFIH1	630.9	843.3	1.7E-10
	MX2	385.0	513.1	3.2E-07
	OAS2	1337.9	1707.1	1.4E-03
	PLSCR1	680.7	875.4	4.8E-12
	RSAD2	382.7	575.8	3.7E-08
	STAT1	4762.1	6824.6	8.9E-12
	STAT2	2027.1	2285.4	2.2E-06
	TAP1	2043.3	2400.8	1.1E-03
	TRAIL	2714.6	3246.5	3.0E-02
	TRAILR2	690.4	785.0	1.1E-05
	XAF1	709.0	847.2	1.3E-02

A similar analysis was carried out after classifying primary breast tumors according to their molecular subtype. Lower expression levels for most of these immune metagenes were also observed in IL-1R8 high luminal A and B molecular subtypes. However, clear differences in the expression level for these immune metagenes were not observed for IL-1R8 high Her2+ and basal-like tumors, possibly due to a small sample size in the case of Her2+ tumors (*n* = 65) and to the lack of IL-1R8 up-regulation in basal-like tumors (Supplementary Tables 3, 4, 5, 6).

These results corroborate our *in vivo and in vitro* observations and indicate that high IL-1R8 expression in primary human breast tumors is associated with a skewed inflammatory response and impairment in the mobilization of protective leukocytes in the TME.

### IL-1R8 expression in clinical samples is associated with a non-T cell-inflamed TME

Solid tumors can be classified into two broad categories according to cellular and molecular characteristics of the TME [37]. T cell–inflamed tumors are characterized by a spontaneous T cell infiltrate, a broad chemokine profile, which supports influx of CD8^+^ effector T cells, and a type I IFN signature indicative of innate immune activation. In contrast, non-T cell-inflamed tumors lack all these characteristics and present an immunosuppressive microenvironment that inhibits the activation of antigen presenting cells and the priming and trafficking of effector T cells [37, 38].

Immune gene signatures based on the expression of T cell specific markers, different chemokines associated with CD8^+^ T cell recruitment [39] and IFN-induced genes have also been used to classify tumors according to their T cell-inflamed phenotype. Since IL-1R8 expressing MMTV-neu mammary tumors present a lower infiltration CD8^+^ T cells and lower levels of intratumoral IFN-γ, we used immune gene signature analysis to investigate whether IL-1R8 expression was associated with a non-T cell-inflamed TME in clinical samples.

**Figure 5:**
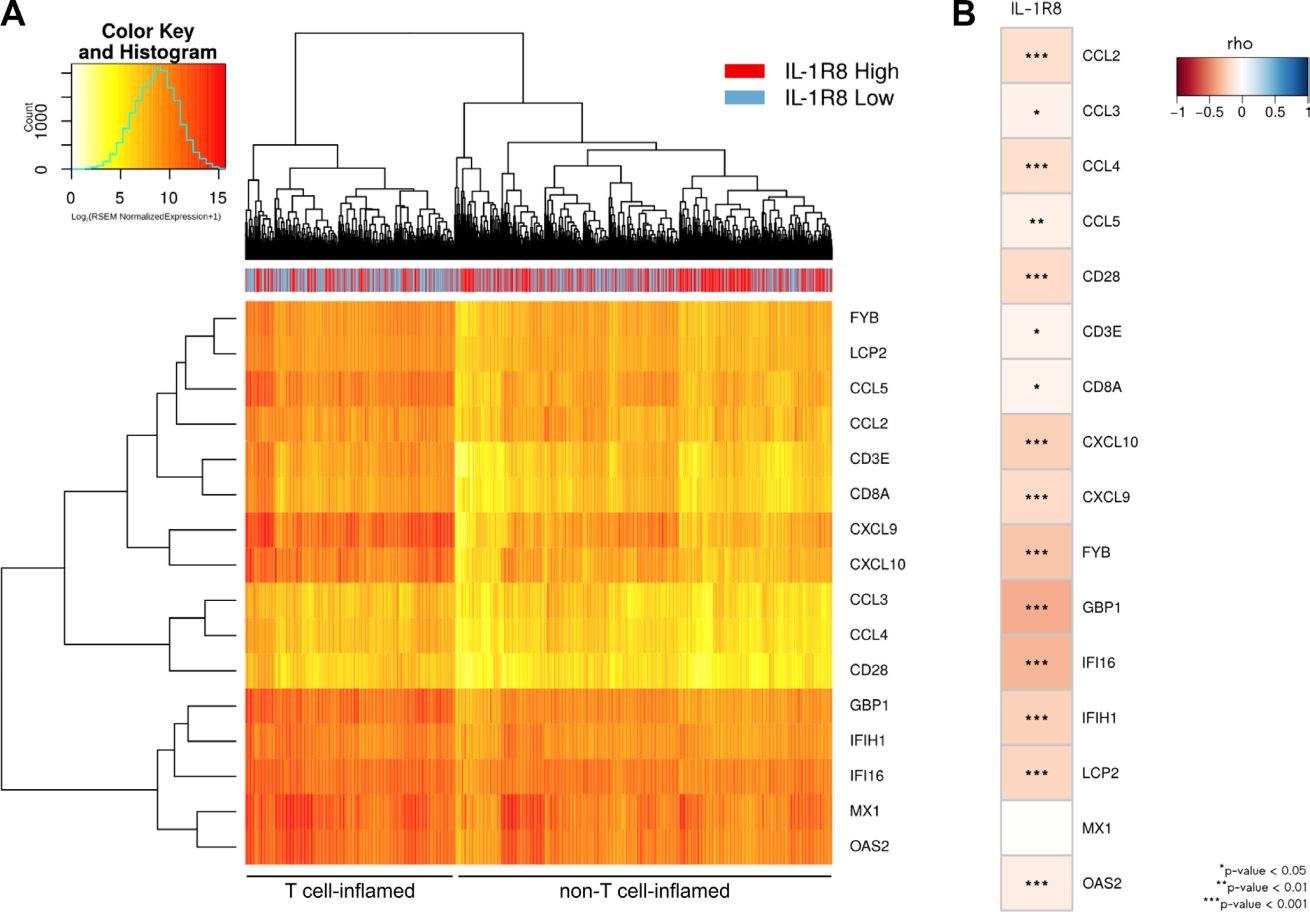
High IL-1R8 expression is associated with a non-T cell inflamed molecular signature in primary breast tumors (**A**) Heat map of 1,102 primary breast tumors clustered in T cell (*N* = 393) and non-T cell (*N* = 709) inflamed groups based on the expression of T cell specific markers (FYB, LCP2, CD3E, CD8A, CD28), different chemokines associated with CD8+ T cell recruitment (CCL5, CCl2, CCl3, CCL4, CXCL9, CXCL10) and IFN-induced genes (IFI16, IFIH1, GBP1, OAS2, MX1). The non-T-cell inflamed cluster is enriched for IL-1R8-high tumors (394/551 or 72%, Chi-square test *P* = 6.758e-07). (**B**) Correlation between IL-1R8 expression and the expression of the 16 genes from the T-cell inflamed signature. Color scale represents Spearman's rho coefficient.

Accordingly, 1,102 primary breast tumors including all major molecular subtypes, were classified into those with a non-T cell-inflamed and those with T cell-inflamed phenotype based of the expression level of T cell specific markers (FYB, LCP2, CD3E, CD8A, CD28), different chemokines associated with CD8^+^ T cell recruitment (CCL5, CCl2, CCl3, CCL4, CXCL9, CXCL10) and IFN-induced genes (IFI16, IFIH1, GBP1, OAS2, MX1). Approximately, 64% of all breast tumors expressed lower expression levels of the 16 T-cell inflamed signature genes (709/1,102) and, noteworthy, the majority of IL-1R8 high tumors were clustered in the group of tumors expressing lower levels of T-cell inflamed signature genes (394/551, 72%, *P* = 6.758e-07, Figure 5A). In addition, a significant inverse correlation between IL-1R8 expression and the expression of all, except one, T cell signature genes was observed (Figure 5B), indicating that IL-1R8 expression is associated with a non-T cell-inflamed TME in breast tumors.

A similar analysis was carried out after classifying primary breast tumors according to their molecular subtype. Lower expression levels for most of these T cell signature genes were also observed for in IL-1R8 high luminal A and B molecular subtypes. However, clear differences in the expression level for these immune metagenes were not observed for IL-1R8 high Her2+ and basal-like tumors, possibly due to a small sample size in the case of Her2+ tumors (*n* = 65) and to the lack of IL-1R8 up-regulation in basal-like tumors (Supplementary Figure 8).

Altogether, these results indicate that high IL-1R8 expression in human breast tumors is associated with a non-T cell inflamed phenotype and may represent a new immune escape mechanism contributing to T cell exclusion and impaired antitumor immunity in this class of tumors.

## DISCUSSION

The activation of innate immune sensors, such as ILRs and TLRs, is an essential mechanism of sterile immunity, playing a critical role in the promotion of pro-tumoral inflammation and mediation of immunosurveillance [10, 40]. Therefore, molecules that negatively regulate their signaling may be exploited by tumors to induce immune tolerance and mitigate host antitumor immune response. Here, we demonstrated for the first time that IL-1R8, a negative regulator of pro-inflammatory signals, is up-regulated in transformed breast epithelial cells and in HER2+ and luminal primary breast cancer molecular subtypes. Most importantly, we demonstrated that its expression contributes to an impaired innate immune sensing and the development of an antitumor immune response.

IL-1R8 negatively regulates ILRs and TLRs signaling by acting as a decoy receptor and by interfering with ILRs dimerization through its Ig domain [13–15]. Recently, it has been demonstrated that, in humans, IL-1R8 can also bind to IL-37, an anti-inflammatory cytokine induced by TLRs and cytokines and a natural suppressor of innate inflammatory response [16, 17]. The binding produces the formation of the tripartite complex involving IL-37/IL-18Rα/IL-1R8, which is necessary for the activation of an anti-inflammatory response. Therefore, in humans, IL-1R8 negatively regulates the innate inflammatory response by acting both as a decoy for TLRs and ILRs signaling and as a co-receptor for IL-37 in the activation of an anti-inflammatory program. In the present work, we have primarily explored the role of IL-1R8 up-regulation in the modulation of ILRs and TLRs signaling. Further studies will be necessary to properly evaluate the implications of IL-37 and IL-1R8 interaction in cancer biology.

IL-1R8 has been previously shown to act as a decoy receptor for ILRs and TLRs in human colon and bladder epithelial cells [23, 41]. In the present work we confirmed IL-1R8 decoy activity in human breast epithelial cells. Using a mammary epithelial cancer cell model, we showed that IL-1R8 expression in transformed cells fine-tuned IL-1-dependent NF-κB activity and the expression of several pro-inflammatory cytokines, including some involved in macrophage polarization (CSF2, IFN-β1 and TNFα) and NK cell activation (IFN-β1 and TNFα) [42–44]. Indeed, using co-culture experiments, we observed that IL-1R8 expression in transformed cells skews macrophage and NK cell activation *in vitro*.

Furthermore, using a murine breast cancer model, we showed that MMTV-neu/IL-1R8^+/+^ mammary tumors grew faster and were more metastatic compared to MMTV-neu/IL-1R8^−/−^ tumors. Although HER2 overexpression is sufficient to drive mammary lesions in MMTV-neu animals, the pace of tumor progression is known to be significantly influenced by tumor-elicited mechanisms of immunosuppression [45, 46]. In line with this evidence, there was a significant increase of tumor-infiltrating leukocytes associated with antitumor immunity, including mature NK cells, DCs and CD8^+^ T cells, in MMTV-neu/IL-1R8^−/−^ mice.

Our *in vivo* results however are in contrast with those obtained with colitis-associated colon-cancer models and lymphocytic leukemia where IL-1R8 has been shown to play a tumor-suppressive role [24–26]. In addition, the aberrant expression of an IL-1R8 splicing isoform, with dominant-negative function in colon tumors, has been recently shown to promote inflammation and colitis-associated colon cancer in mouse and humans [47]. The dual role of inflammation in cancer is well known and several lines of evidence indicate that inflammatory and immune mechanisms in cancer are tissue- and organ specific [48, 49]. Therefore, it is not surprising that IL-1R8 can act as a tumor suppressor in models of cancer strongly dependent on inflammation, while having a tumor promoting effect by fine-tuning inflammation and avoiding detection and eradication by the immune system in other tumor types. In line with our results, IL-1R8 up-regulation in prostate cancer has been recently associated with worse prognosis and can predict biochemical recurrence after prostatectomy in low-grade prostate cancer patients [50].

In accordance with our *in vitro* experiments using IL-1R8-knockdown cancer cells, we observed that MMTV-neu/IL-1R8^−/−^ TAMs showed reduced expression of markers associated with an M2-like phenotype, in particular CD206, YM1, IL-10 [43]. TAMs usually acquire a skewed M2-like phenotype oriented to tumor promotion and it has been reported that Th2 CD4^+^ T cells promote breast tumor progression and metastasis by educating TAMs to produce pro-angiogenic and pro-metastatic factors [51]. Therefore, the results showing that M1-macrophage polarization, directly induced by MMTV-neu/IL-1R8^−/−^ tumors or indirectly induced by leukocytes recruited in the tumor, such as NK cells, DCs and CD8^+^ T lymphocytes, may also account for reduced tumor growth and metastasis observed in MMTV-neu/IL-1R8^−/−^ mice.

Notably, the protected phenotype observed in MMTV-neu/IL-1R8^−/−^ mice was not reversed when mice were transplanted with IL-1R8^+/+^ bone-marrow cells, supporting an important role played by IL-1R8 expression in non-hematopoietic cells. Since besides leukocytes, cells which express highest levels of IL-1R8 are epithelial cells [13], we hypothesize that the observed effects on tumor growth and progression can be directly attributed to IL-1R8 expression in tumor cells, which might be sufficient to influence the inflammatory and immune responses occurring within the TME.

Using immune gene signature analysis, we confirmed that high IL-1R8 expression in primary breast tumors is significantly associated with impaired innate immune sensing and the mobilization of protective leukocytes. Using the same approach, we have also shown that the majority of primary breast tumors displayed a non-T cell inflamed phenotype and that high IL-1R8 expression in primary breast tumors is associated with lower expression levels of T-cell inflamed signature genes.

Increasing evidence support that current immunotherapies such as checkpoint blockade are predominantly effective in patients with a pre-existing T cell-inflamed tumor microenvironment [52–54]. Understanding the molecular mechanisms leading to T cell exclusion and to resistance to T cell-based immunotherapies would improve patient selection and allow the development of novel treatment modalities, expanding the fraction of patients benefiting from current immunotherapies. In this context, further studies to address a putative role for IL-1R8 expression as a prognostic and predictive marker for immunotherapy in breast cancer are necessary and are worth undertaking.

Interestingly, pre-clinical studies have demonstrated that the use of different agents to induce tissue-based inflammation and boost innate immunity, including activation of the Stimulator of Interferon Genes (STING) and local irradiation, can restore T cell trafficking and favorably alter the TME [55, 56]. Based on our findings, we propose that IL-1R8 expression in breast tumors represents a new immunomodulatory mechanism leading to a dysregulated inflammatory response and impaired antitumor immunity. Our findings therefore have important therapeutic implications and strategies to block IL-1R8 activity directly or through combined therapies as a way to restore innate immune system activation and T cell trafficking in breast cancer TME should be further explored.

## MATERIALS AND METHODS

### Cell lines and IL-1R8 knockdown

The human mammary epithelial cell line HB4a and its variant HB4a – C5.2 were kindly donated by Dr. Michael O'Hare (Institute of Cancer Research, Sutton, UK). The human natural killer cell line (NKL) and the human leukocytic monocyte cell line (THP-1) were obtained from American Type Culture Collection (Manassas, VA, USA). HB4a-C5.2 cells were transfected with two shRNA constructs for IL-1R8 or the empty vector (TrifectaTM Kit – IDT, Coralville, IA, USA).

### Co-culture experiments

Cells were treated with 5 ng/mL of IL-1β (R&D Systems, Minneapolis, MN, USA). After 24 h, the culture supernatant was collected and stored at −80°C. THP-1 cells were cultured for 24 h with supernatants or 25 ng/mL LPS (M1-like control) or 30 ng/mL IL-4 (M2-like control). Cells were stained with anti-CD206 (eBioscience, San Diego, CA, USA), anti-CD86 (BD Biosciences, San Jose, CA, USA), anti-CD14 (BD Biosciences) and analyzed using FACScantoII (BD Biosciences). For co-culture assays with NK cells, human transformed mammary cell lines were seeded and allowed to adhere to the plate for 24 h. NKL cells were added in their complete medium and cells were co-cultured for 4h. IFN-γ secretion was measured by ELISA.

### Animal model

F3 MMTV-neu/IL-1R8^+/+^ and MMTV-neu/IL-1R8^−/−^ female mice were sacrificed at 24 weeks of age or once their biggest mammary tumor reached 500 mm^3^. For bone marrow transplantation, 3-weeks old MMTV-neu/IL-1R8^−/−^ mice were lethally irradiated with a total dose of 900 cGy. Mice were then injected in the retro-orbital plexus with 5 × 10^6^ nucleated bone marrow cells from IL-1R8^+/+^ or IL-1R8^−/−^ donors. All experimental procedures were performed according to the Italian animal care and ethics legislation and had been approved by the Italian Ministry of Health.

### FACS analysis

The composition of tumor infiltrate was determined by flow cytometry using different combinations of the following antibodies: CD45-BV605, CD11b-BV421; Ly6G-PE-CF594; Ly6C-FITC (Clone AL-21); F4/80-PECy7; CD11c-PE; CD3e-FITC; CD4-Alexa700; CD8-PE; CD19-APC-H7; CD49b(DX5)-APC and MHCII-PercpCy5.5 from BD Bioscience, eBioscience or BioLegend (San Diego, CA, USA). Cell viability was determined by Aqua LIVE/Dead-405 nm staining (Invitrogen). Cells were analyzed on LSR Fortessa (BD Bioscience).

### *In silico* analysis of gene expression, metagenes and immune gene signature analyses

Breast invasive carcinoma Level 3 RNA-Seq data were downloaded from TCGA Portal (https://tcga-data.nci.nih) and molecular subtypes were classified as described [57]. Upper quartile normalized RSEM counts were used to estimate expression levels of IL-1R8 across 1,102 tumor samples and 113 normal samples. 792 out of those 1,102 samples, for which molecular classification was available, were used to compare IL-1R8 expression levels between each subtype. For the metagenes and immune gene signature analyses, tumor samples were classified into: “IL-1R8-high”, if presenting IL-1R8 expression greater than the median; and “IL-1R8-low” if presenting IL-1R8 expression equal or less than the median.

## SUPPLEMENTARY MATERIALS FIGURES AND TABLES




