# Supplementary material for: High IL-1R8 expression in breast tumors promotes tumor growth and contributes to impaired antitumor immunity
Source: Oncotarget. 2017 May 9;8(30):49470–83. doi: 10.18632/oncotarget.17713 (PMC5564782; doi:10.18632/oncotarget.17713)
Supplement: Supplementary file 1 [file oncotarget-08-49470-s001.pdf]

High IL-1R8 expression in breast tumors promotes tumor growth and contributes to impaired antitumor immunity

SUPPLEMENTARY MATERIALS

MATERIALS AND METHODS

Cell lines

The human epithelial mammary cell lines HB4a and C5.2 cells lines were kindly donated by Dr. Michael O'Hare after signing a Material Transfer Agreement with The Ludwig Institute for Cancer Research. The parental HB4a cell line was established by Stamps et al, 1994 [1] and the ErbB-2 overexpressing variant C5.2 was derived by Harris et al, 1999 [2] from HB4a by stable co-transfection with full-length normal human ErbB-2 cDNA under the control of the MMTV-LTR promoter and SV40 polyadenylation signals. Cells were kept using low passage numbers (maximum 12 passages) and the lowest-passage aliquot was used to start fresh cultures. Cells were also routinely checked for Mycoplasma contamination. Since no reference DNA fingerprint was established for these cells lines, cell identity was confirmed by morphological characterization, proliferation rate and ErbB-2 copy number genotyping. DNA fingerprinting was used to confirm the parental origin of the C5.2 cell line and to check for cross-contamination with other cell lines available in the laboratory. HB4a and C5.2 cells were cultured using RPMI medium (Life Technologies) supplemented with 10% fetal bovine serum (FBS) (Cultilab), 5 mg/mL insuline (Sigma-Aldrich), 5 mg/mL hydrocortisone and 1% L-glutamine (Sigma-Aldrich). The human natural killer cell line (NKL) and the human leukocytic monocyte cell line (THP-1) were obtained by ATCC and were cultured in RPMI-1640 medium supplemented with 10% fetal bovine serum (FBS), penicillin (100 U/mL), streptomycin (100 μg/mL), and 200 U/mL of human recombinant IL-2 (Biolegend) or 0.05 mM 2-mercaptoethanol and 1% L-glutamine (Sigma-Aldrich), respectively. Cells were kept at 37ºC in a humidified atmosphere containing 5% CO_2.

Generation of knockdown cells

HB4a-C5.2 cells were transfected with shRNA for SIGIRR/IL-1R8 (5′- GGAGAUAAACGACGGG AAGCUCUAC-3′ and 3′- CACCUCUAUUUGCUG CCCUUCGAGAUG-5′) or the empy vector, TrifectaTM Kit (IDT – Integrated DNA Technologies), using Lipofectamine RNAi Max (Invitrogen) with a reverse transfection protocol and clones were selected with 2 ug/mL Puromycin (Promega). For the sake of simplicity, results obtained for the wild-type cells and cells transfected with a control shRNA vector (EV1) were grouped throughout the text and presented as the HB4aHER2+, whereas results obtained for two independent IL-1R8-shRNA clones (63.2 and 7B13) derived from different shRNAs constructs were grouped and presented as $HB4a^{HER2+/IL1R8KD}$.

Immunoblot analysis and EMSA

Proteins were extracted using lysis buffer (50 mM Tris–HCl pH 7.4, 1% Nonidet P-40, 0.25% sodium deoxycholate, 150 mM NaCl, 1 mM EDTA, 1 mM PMSF and cocktail of protease and phosphatase inhibitors (Sigma–Aldrich). Quantified total protein extracts were resolved on a 10% to 12% sodium dodecyl sulfate–polyacrylamide electrophoresis gel, transferred to nitrocellulose membranes (GE Healthcare Biosciences) and probed with antibody anti-mSIGIRR/IL-1R8 Ab (1:200; ProSci; #3367), anti-IkB (1:500; Santa Cruz Biotechnology; #PAB C-21), anti-β-tubulin (1:2000; Cell Signalling Technology; #2146) or anti-mVinculin (1:200; Sigma–Aldrich; V9131) antibodies overnight in 0.1% Tween-20 with 5% milk. Membranes were incubated for 60 min at room temperature in 0.1% Tween-20 with the secondary HRPT-conjugated antibody (1:5000; GE Healthcare Biosciences; #NA931v and #NA934v). Reactive proteins were visualized with the ECL Western Blotting Detection System (GE Healthcare Biosciences) according to the manufacturer's recommendations. The nuclear protein extract and the Electrophoretic mobility shift assay (EMSA) for NF-kB was performed as previously described [3].

Gene expression analysis

Total RNA was extracted using Trizol (Life Technologies). Reverse transcription (RT) was carried out with 2 μg DNA-free total RNA using Superscript II reverse transcriptase (Life Technologies). Quantitative PCR (qRT-

PCR) was performed using a 7300 Real-time PCR System and SYBR Green (Life Technologies). Relative expression levels were calculated as described [4]. The list of primers used is found in Supplementary Table 7.

Preparation of conditioned-medium and co-culture experiments

Cells were seeded (4×10^5 cells/well) in 6-well plates and treated with 5 ng/mL of IL-1β (Recombinant Human IL-1β - R&D Systems) in RPMI supplemented with 1% fetal bovine serum (FBS). After a period of 24 h, the medium was collected, centrifuged at 12000 x g at 4ºC to remove cell debris and stored at –80ºC until use. For the macrophage polarization assay, the human monocytic cell line THP-1 was differentiated to macrophage-like cells as previously described [5]. Cells were then cultured for 24 h with the respective treatments diluted in RPMI medium 10×, LPS 25 ng/mL (M1-like control) and IL-4 30 ng/mL (M2-like control). Cells were then stained with anti-CD206 (eBioscience), anti-CD86 (BD Biosciences), anti-CD14 (BD Biosciences) and analyzed using FACScantoII (BD Biosciences). For the co-culture assay with NK cells, the human transformed mammary cell lines were seeded in 24-well plates (5×10^4 cells/well) and were allowed to adhere to the plate for 24 h. Medium was changed and NKL cells (5×10^5 cells per well) were added in their complete medium. Cells were co-cultured for 4 h at 37°C. Cell supernatants were then collected, centrifuged for 15 min at 12000 G at 4ºC to remove cell debris and kept at –80ºC for further analysis of IFN-γ secretion by ELISA.

Animals

Mice used were on a mixed BALB/c and C57BL/6J genetic background (F3), obtained by crossing MMTV-neu on a BALB/c background and IL-1R8-deficient mice on a C57Bl/6J background. IL-1R8-deficient mice were generated as described [6]. Procedures involving animals handling and care were conformed to protocols approved by the Humanitas Clinical and Research Center (Rozzano, Milan, Italy) in compliance with national (D.L. N.116, G.U., suppl. 40, 18-2-1992 and N. 26, G.U. March 4, 2014) and international law and policies (EEC Council Directive 2010/63/EU, OJ L 276/33, 22-09-2010; National Institutes of Health Guide for the Care and Use of Laboratory Animals, US National Research Council, 2011). The study was approved by the Italian Ministry of Health (approval n. 43/2012-B, issued on the 08/02/2012). Animal's sample size was defined on the basis of past experience on cancer models, in order to detect differences of 20% or grater between the groups (10% significance level and 80% power). All efforts were made to minimize the number of animals used and their suffering. In most *in vivo* experiments, the investigators were blinded for the genotype of the experimental groups. Most *in vivo* experiments were replicated at least twice. F3 MMTV-neu/IL-1R8$^{+/+}$ and MMTV-neu/IL-1R8$^{-/-}$ female mice were used in two experimental groups composed by mice sacrificed at 24 weeks of age and mice sacrificed once their biggest mammary tumor reached 500 mm^3 of volume. Tumor volume was determined by the formula: $V = 1⁄2 (D \times d^2)$ (considering D the larger diameter and d the minor diameter) only in tumor-bearing animals. Mammary fat pads were checked weekly for the presence of tumors and, when present, measured using a calliper.

Histopathology and immunohistochemistry

At least eight samples from PFA-fixed, paraffin-embedded mouse tumor tissues were analyzed for each condition. Consecutive sections from the middle of the tissue were used for histological examination in each mouse. Sections for histological analysis were stained with Haematoxylin-Eosin (H&E) and examined blindly by a pathologist (M.N.). Paraffin-embedded tissue sections were mounted on Super-frost slides, dewaxed in xylene and rehydrated in ethanol. Endogenous peroxidase was blocked for 20 min in 90% ethanol containing 2% H2O2. Sections were then pretreated in a microwave oven (two cycles for 3 min each at 800 W, in 0.25 mM EDTA buffer). Unspecific sites were blocked with Rodent Block M (Biocare Medical) 30 min and tissues were incubated for two hours with affinity-purified anti-CD45 (Rat anti-Mouse, R&D Systems), anti-IL-1R8 (Goat anti-mouse, R&D Systems) and anti-F4/80 (Rat anti-Mouse, AbD Serotec) primary antibodies in PBS supplemented with BSA (1%) and NP40 (0.02%). Sections were then washed with washing buffer (PBS$^{-/-}$ + 0,05% Tween 20) and incubated with a two-steps detection system Rat-on-Mouse HRP-Polymer Kit (Biocare medical). After washing, slides were developed with DAB (3,30-diaminobenzidine) (Biocare Medical) and counterstained with Hematoxilyn. Tissue slides were then analysed using a computer-aided image analysis software (Olympus Dotslide).

ELISA

Tumors were homogenized in 1ml PBS^{2+} containing protease inhibitors (Complete®-EDTA-free; Roche Diagnostics) and PMSF (1mM). Tissue homogenates were centrifuged at 14000 rpm for 30 min at 4°C and supernatants were stored at –80°C for cytokine analysis. The presence of IL-1β, IL-10, IL-12, IFN-γ, VEGF, CSF2 and CCL2 were measured by ELISA (R&D DuoSet ELISA Development System) according to manufacturer's instructions.

Purification of tumor-associated macrophages (TAMs)

CD11b$^+$ cells from tumors were MACS enriched according to manufacturer's instructions (Miltenyi Biotec). Purity of CD11b$^+$ cells was about 90% as determined by FACS. CD11b+ cells were stained with LIVE/DEAD® Fixable Aqua Dead Cell Stain Kit (Life Technologies)

to exclude non-viable cells. CD11b+ cells were then prestained with FcBlock and stained with CD11b-BV421; Ly6C-FITC; Ly6G-PE; MHCII- Percp-Cy5.5 and NK1.1-PE as described [7] and sorted on a FACSAria cell sorter (BD Bioscience). Antibodies anti-NK1.1 and anti-Ly6G were used to exclude NK cells and granulocytes. Purity of sorted $CD11b^{+}Ly6G^{-}Ly6C^{-}NK1.1^{-}$ $MHCII^{low}$ and $CD11b^{+}Ly6G^{-}Ly6C^{-}NK1.1^{-}$ $MHCII^{high}$ was > 98%. Resulting cells were processed for mRNA extraction.

Quantitative PCR of purified TAMs

Total RNA was extracted using Trizol reagent (Invitrogen) following the manufacturer's recommendations. RNA was further purified using RNeasy Min-elute RNA isolation kit (QIAGEN). cDNA was synthesized using 500 ng of total RNA by reverse transcription using High Capacity cDNA archive kit (Applied Biosystems) and quantitative real-time PCR was performed using the SybrGreen PCR Master Mix (Applied Biosystems) in a ViiA™ 7 Real-Time PCR System (Life technologies). Data were analyzed with the D2CT method and normalized based on 18S expression determined in the same sample. Analysis of all samples was performed in triplicate. Primers were designed according to the published sequences and listed as follows in Supplementary Table 3.

Generation of bone marrow chimeras

3-weeks old MMTV-neu/IL-1R8$^{-/-}$ mice (n = 10 per group) were lethally irradiated with a total dose of 900 cGy. Mice were then injected in the retro-orbital plexus with 5×10^6 nucleated bone marrow cells obtained by flushing of the cavity of a freshly dissected femur from IL-1R8$^{+/+}$ or IL-1R8$^{-/-}$ donors. Recipient mice received gentamycin (0.8 mg/ml in drinking water) starting 7 days before irradiation and maintained during 2 weeks. Mice were then followed until 24 weeks of age and the presence of mammary tumors was assessed weekly.

In silico analysis of gene expression, metagenes and immune gene signature analyses

Breast invasive carcinoma Level 3 RNA-Seq data were downloaded from TCGA Portal (https://tcga-data.nci.nih) and molecular subtypes were classified as described [8]. Upper quartile normalized RSEM counts were used to estimate expression levels of IL-1R8 across 1,102 tumor samples and 113 normal samples. 792 out of those 1,102 samples, for which molecular classification was available, were used to compare IL-1R8 expression levels between each subtype (136 Basal samples; 65 Her2+ samples; 415 Luminal A samples; 176 Luminal B samples) and normal breast samples. Statistical differences of IL-1R8 transcription levels between subtypes and normal samples were calculated using Wilcoxon rank-sum test. For the metagenes and immune gene signature analyses, tumor samples were classified into: "IL-1R8-high", if presenting IL-1R8 expression greater than the median; and "IL-1R8-low" if presenting IL-1R8 expression equal or less than the median. Expression levels of selected immune-related genes in "IL-1R8-high" and "IL-1R8-low" samples were compared using Wilcoxon rank-sum test. For the T-cell signature analysis, we sub-selected 16 genes: T cell specific markers (FYB, LCP2, CD3E, CD8A, CD28), chemokines associated with CD8+ T cell recruitment (CCL5, CCl2, CCl3, CCL4, CXCL9, CXCL10); and IFN-induced genes (IFI16, IFIH1, GBP1, OAS2, MX1) and performed a hierarchical clustering analysis based on their expression levels. Chi-square test was carried out to evaluate the enrichment for IL-1R8-high samples. Correlation between the expression levels of IL-1R8 and selected genes was evaluated by Spearman's rank correlation. P-values were adjusted for multiple comparisons using Benjamini-Hochberg method.

Statistical analysis

All values were expressed as mean ± SEM of biological replicates. Two-sided unpaired or paired Student's t test (normal distribution, equal variance) was used as specified. In case variance among groups was statistically different, Mann-Whitney (non-parametric) test was used, as indicated. ROUT test was applied to exclude outliers and experiments were repeated at least twice. Significant differences were considered at $^{*}P < 0.05$, $^{**}P < 0.01$ and $^{***}P < 0.001$.

REFERENCES

1. Stamps AC, Davies SC, Burman J, O'Hare MJ. Analysis of proviral integration in human mammary epithelial cell lines immortalized by retroviral infection with a temperature-sensitive SV40 T- antigen construct. Int J Cancer. 1994; 57:865–74. doi: 10.1002/ijc.2910570616.
2. Harris RA, Eichholtz TJ, Hiles ID, Page MJ, O'Hare MJ. New model of erbB-2 over-expression in human mammary luminal epithelial cells. Int J Cancer. 1999; 80:477–84. doi: 10.1002/(SICI)1097-0215(19990129)80:3<477::AID-IJC23>3.0.CO;2-W.
3. Stossi F, Madak-Erdoğan Z, Katzenellenbogen BS. Corticosterone modulates noradrenaline-induced melatonin synthesis through inhibition of nuclear factor kappa B. J Pineal Res. 2005; 38:182–8. doi: 10.1111/j.1600-079X.2004.00191.x.
4. Pfaffl MW, Pfaffl MW. A new mathematical model for relative quantification in real-time RT-PCR. Nucleic Acids Res. 2001; 29:e45. doi: 10.1093/nar/29.9.e45.
5. Fabio Stossi, Zeynep Madak-Erdoğan and BSK. Macrophage-Elicited Loss of Estrogen Receptor Alpha in Breast Cancer Cells via Involvement of MAPK and c-Jun at the ESR1 Genomic Locus. Oncogene. 2012; 29:997–1003. doi: 10.1016/j.biotechadv.2011.08.021.Secreted.

6. Garlanda C, Riva F, Polentarutti N, Buracchi C, Sironi M, De Bortoli M, Muzio M, Bergottini R, Scanziani E, Vecchi A, Hirsch E, Mantovani A. Intestinal inflammation in mice deficient in Tir8, an inhibitory member of the IL-1 receptor family. Proc Natl Acad Sci USA. 2004; 101:3522–6. doi: 10.1073/pnas.0308680101.
7. Laoui D, Van Overmeire E, Conza G Di, Aldeni C, Keirsse J, Morias Y, Movahedi K, Houbracken I, Schouppe E, Elkrim Y, Karroum O, Jordan B, Carmeliet P, et al. Tumor hypoxia does not drive differentiation of tumor-associated macrophages but rather fine-tunes the M2-like macrophage population. Cancer Res. 2014; 74:24–30. doi: 10.1158/0008-5472.CAN-13-1196.
8. Ciriello G, Gatza ML, Beck AH, Wilkerson MD, Rhie SK, Pastore A, Zhang H, McLellan M, Yau C, Kandoth C, Bowlby R, Shen H, Hayat S, et al. Comprehensive Molecular Portraits of Invasive Lobular Breast Cancer. Cell. 2015; 163:506–19. doi: 10.1016/j.cell.2015.09.033.

Supplementary Table 1: Histopathological features of MMTV-neu mammary tumors

**Histopathological features**	**MMTV-neu/IL-1R8$^{+/+}$(*n* = 6)**	**MMTV-neu/IL-1R8$^{-/-}$ (*n* = 5)**
Tumor growth		
Expansive, n (%)	2 (33.3)	5 (100)
Infiltrative, n (%)	4 (66.6)	0 (0)
	++ (16.7)	++ (0)
Tissue necrosis (%)	+ (83.3)	+ (60)
	No (0)	No (40)
	+++ (16.7)	+++ (0)
Cellular atypia (%)	++ (32.3)	++ (20)
	+ (50)	+ (80)
Number of mitosis (per field)	2.5 ± 0.5^{NS}	1.2 ± 0.5^{NS}

Supplementary Table 2: Expression levels of 68 immune-related genes in BRCA samples. See Supplementary_Table_2

Supplementary Table 3: Expression levels of immune-related genes in IL-1R8-high and IL-1R8-low in Basal breast tumors

		IL-1R8 High	IL-1R8 Low	adj-P
**T-Cell transcripts**	CD28	77.8	63.4	5.0E-01
	CD3G	37.9	38.0	4.9E-01
	CD8A	291.1	176.4	1.2E-01
	CD8B	117.2	56.3	1.2E-01
	FYB	578.8	503.1	7.8E-01
	ICOS	59.8	46.4	4.4E-01
	LCP2	490.6	416.3	5.0E-01
	LTA	32.8	19.9	1.6E-01
**CD8+ T-cell Chemokines**	CCL2	660.8	551.2	5.0E-01
	CCL3	130.5	106.4	5.0E-01
	CCL4	192.5	126.4	5.0E-01
	CCL5	1403.4	933.7	1.4E-01
	CXCL9	2071.0	2023.3	4.9E-01
	CXCL10	2200.7	992.1	4.9E-01
**IFN-induced genes**	EIF2AK2	772.9	831.5	4.4E-01
	GBP1	2825.7	2491.2	7.8E-01
	IFI16	3299.2	2265.0	4.4E-01
	IFIH1	1424.5	1447.7	7.8E-01
	MX2	1033.0	775.3	4.9E-01
	OAS2	2000.5	1854.7	7.8E-01
	PLSCR1	1395.1	1504.8	5.0E-01
	RSAD2	711.7	661.3	8.4E-01
	STAT1	8961.9	9234.9	7.3E-01
	STAT2	2277.1	2286.7	8.6E-01
	TAP1	5590.7	4137.5	4.9E-01
	TRAIL	2057.0	1942.4	8.6E-01
	TRAILR2	873.6	888.9	9.5E-01
	XAF1	1432.5	1151.2	4.4E-01

Supplementary Table 4: Expression levels of immune-related genes in IL-1R8-high and IL-1R8-low in HER2+ breast tumors

		IL-1R8 High	IL-1R8 Low	adj-P
T-Cell transcripts	CD28	80.6	67.0	4.5E-01
	CD3G	40.4	26.4	3.8E-01
	CD8A	214.7	139.2	1.5E-01
	CD8B	62.0	33.9	1.5E-01
	FYB	633.1	548.4	7.4E-01
	ICOS	57.7	40.5	2.4E-01
	LCP2	543.1	412.2	6.6E-01
	LTA	27.8	12.5	1.9E-01
CD8+ T-cell Chemokines	CCL2	455.1	309.5	5.5E-01
	CCL3	136.5	102.5	4.2E-01
	CCL4	132.0	89.6	2.9E-01
	CCL5	1208.1	487.4	1.2E-01
	CXCL9	2472.4	1523.5	3.8E-01
	CXCL10	1518.5	918.2	4.0E-01
IFN-induced genes	EIF2AK2	592.4	604.1	6.1E-01
	GBP1	2134.9	1859.4	5.6E-01
	IFI16	1900.9	1701.8	8.2E-01
	IFIH1	862.1	798.3	4.8E-01
	MX2	611.5	401.9	2.5E-01
	OAS2	1980.1	1408.0	5.8E-01
	PLSCR1	739.9	634.0	6.6E-01
	RSAD2	669.4	615.0	9.6E-01
	STAT1	11330.1	8090.3	4.5E-01
	STAT2	2452.8	2373.6	9.6E-01
	TAP1	4407.6	2212.6	1.5E-01
	TRAIL	4640.7	3474.8	9.6E-01
	TRAILR2	527.6	654.8	1.5E-01
	XAF1	879.4	638.0	2.3E-01

Supplementary Table 5: Expression levels of immune-related genes in IL-1R8-high and IL-1R8-low in Luminal A breast tumors

		IL-1R8 High	IL-1R8 Low	adj-P
**T-Cell transcripts**	CD28	40.1	57.5	5.5E-03
	CD3G	15.4	26.8	5.5E-03
	CD8A	135.3	162.0	9.2E-01
	CD8B	34.2	39.3	9.5E-01
	FYB	317.3	470.8	1.3E-04
	ICOS	12.7	16.0	3.9E-01
	LCP2	275.4	363.2	6.2E-03
	LTA	7.3	8.2	9.4E-01
**CD8+ T-cell Chemokines**	CCL2	297.6	339.5	2.6E-01
	CCL3	80.5	80.6	9.4E-01
	CCL4	68.0	76.9	3.9E-01
	CCL5	437.0	443.3	8.7E-01
	CXCL9	268.2	328.3	5.3E-01
	CXCL10	268.2	328.3	5.3E-01
**IFN-induced genes**	EIF2AK2	408.0	534.3	1.4E-06
	GBP1	666.0	851.7	1.2E-02
	IFI16	1437.8	1923.4	3.8E-06
	IFIH1	602.8	714.5	3.1E-02
	MX2	359.5	412.8	3.9E-01
	OAS2	1229.3	1481.1	1.3E-01
	PLSCR1	638.8	706.5	3.6E-01
	RSAD2	344.1	464.8	9.2E-02
	STAT1	4446.6	5154.9	1.4E-02
	STAT2	1940.2	2198.3	1.1E-03
	TAP1	1924.3	1680.3	2.8E-01
	TRAIL	2868.2	4252.9	1.4E-04
	TRAILR2	751.4	792.7	1.4E-01
	XAF1	598.1	644.9	6.2E-01

Supplementary Table 6: Expression levels of immune-related genes in IL-1R8-high and IL-1R8-low in Luminal B breast tumors

		IL-1R8 High	IL-1R8 Low	adj-P
**T-Cell transcripts**	CD28	45.7	58.5	3.6E-01
	CD3G	14.9	19.8	4.5E-01
	CD8A	115.1	124.2	7.1E-01
	CD8B	28.7	24.8	6.9E-01
	FYB	365.7	387.9	1.6E-01
	ICOS	18.2	27.3	4.9E-01
	LCP2	301.7	358.5	5.1E-01
	LTA	8.7	10.6	6.5E-01
**CD8+ T-cell Chemokines**	CCL2	257.2	299.1	5.1E-01
	CCL3	87.2	91.5	6.9E-01
	CCL4	71.6	90.1	5.2E-01
	CCL5	389.6	373.3	9.3E-01
	CXCL9	524.9	661.1	5.2E-01
	CXCL10	401.3	585.4	2.1E-01
**IFN-induced genes**	EIF2AK2	587.5	769.0	3.0E-02
	GBP1	677.1	1104.2	6.0E-02
	IFI16	1148.2	1433.4	2.2E-02
	IFIH1	767.1	1058.6	6.9E-02
	MX2	364.9	509.5	3.6E-01
	OAS2	1375.4	2374.3	1.6E-01
	PLSCR1	702.9	910.3	1.6E-01
	RSAD2	483.9	905.3	6.0E-02
	STAT1	5893.3	9081.9	3.5E-02
	STAT2	2046.1	2312.9	1.6E-01
	TAP1	2263.8	2606.7	9.3E-01
	TRAIL	1874.1	3119.8	1.7E-02
	TRAILR2	551.6	591.6	5.0E-01
	XAF1	817.5	1089.9	4.5E-01

Supplementary Table 7: List of primers for gene-expression analysis

Gene name	Forward (5′-3′)	Reverse (5′-3′)
**SIGIRR/IL-1R8**	GTCTCAACGTGCTGCTCTGGTA	TCGTAGAGCTTCCCGTCGTTTA
**IL-6**	TGCAGATGAGTACAAAAGTCCTGT	GTGGTTATTGCATCTAGATTCTTTGC
**IL-8**	GCAGAGGGTTGTGGAGAAGTTT	TTGGATACCACAGAGAATGAATTTTT
**TNFα**	CTGGCCCAGGCAGTCAGAT	GGTTTGCTACAACATGGGCTACA
**IFN-β1**	GCAATTGAATGGGAGGCTTG	ATAGATGGTCAATGCGGCGT
**CSF2**	TGATGGCCAGCCACTACAAG	GGGTTGCACAGGAAGTTTCC
**CSF3**	CTCCAGGAGAAGCTGGCAGG	GGCCATTCCCAGTTCTTCCA
**HPRT**	TGACACTGGCAAAACAATGCA	GGTCCTTTTCACCAGCAAGCT
**Chil3 (Ym1)**	TCTGGGTACAAGATCCCTGAA	TTTCTCCAGTGTAGCCATCCTT
**IFN-γ**	TCAAGTGGCATAGATGTGGAAGAA	TGGCTCTGCAGGATTTTCATG
**IL-10**	GGTTGCCAAGCCTTATCGGA	ACCTGCTCCACTGCCTTGCT
**STAB1**	CCCTCCTTCTGCTCTGTGTC	CAAACTTGGTGTGGATGTCG
**MCR1 (CD206)**	TGGCATGTCCTGGAATGAT	CAGGTGTGGGCTCAGGTAGT
**TNFα**	AAGAGGCACTCCCCCAAAAG	CTTGGTGGTTTGCTACGACG
**18s/18S**	ACTTTCGATGGTAGTCGCCGT	CCTTGGATGTGGTAGCCGTTT

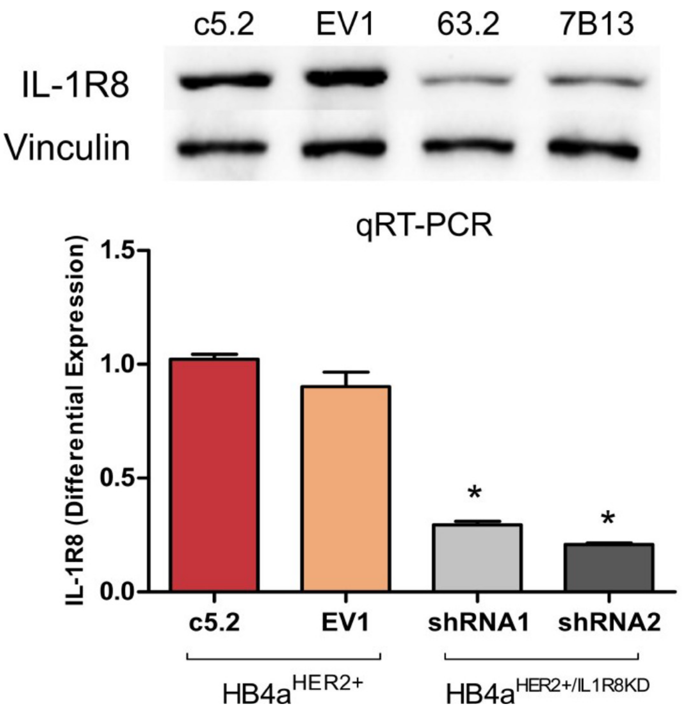

Supplementary Figure 1: Generation of IL-1R8 Knockdown cells by shRNA in HB4a^{HER2+} cells. IL-1R8 protein expression by western-blot (upper part) and mRNA expression by qRT-PCR (lower part) in IL-1R8-knockdown clones (63.2 and 7B13). HB4a-c5.2 control cells and cells transfected with the empty-vector (EV1) were grouped as HB4a^{HER2+}. IL-1R8-shRNA independent knockdown clones derived from different shRNA constructs were grouped as HB4a$^{HER2+/IL1R8KD}$. Error bars indicate the variation between the means of three independent experiments.

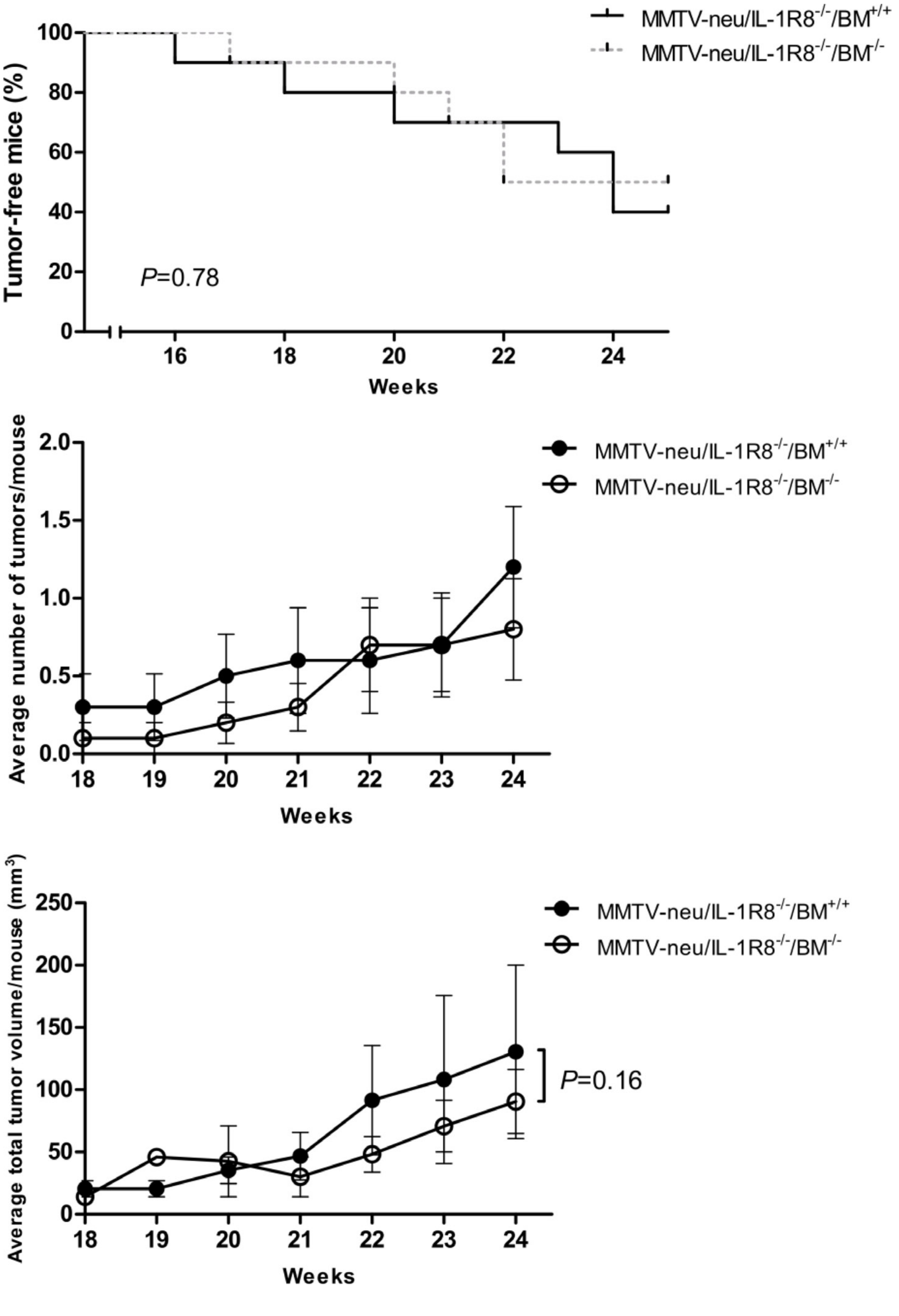

Supplementary Figure 2: Protective phenotype of MMTV-neu/IL-1R8$^{-/-}$ mice is not reversed with IL-1R8+/+ bone-marrow transplantation. Transplant of IL1-R8+/+ bone-marrow cells (BM) does not revert protected MMTV-neu/IL-1R8$^{-/-}$ phenotype. (**A**) Kaplan–Meier analysis of tumor-free survival of irradiated MMTV-neu/IL-1R8$^{-/-}$ transplanted with IL1-R8+/+ (n = 10) or IL1-R8−/− (n = 10) BM. Log-rank test, P = 0.78. (**B**) Average number of mammary tumors per mouse over time (weeks); (**C**) Average total tumor volume per mouse over time (weeks). (B and C), Unpaired or paired Student's t test, respectively.

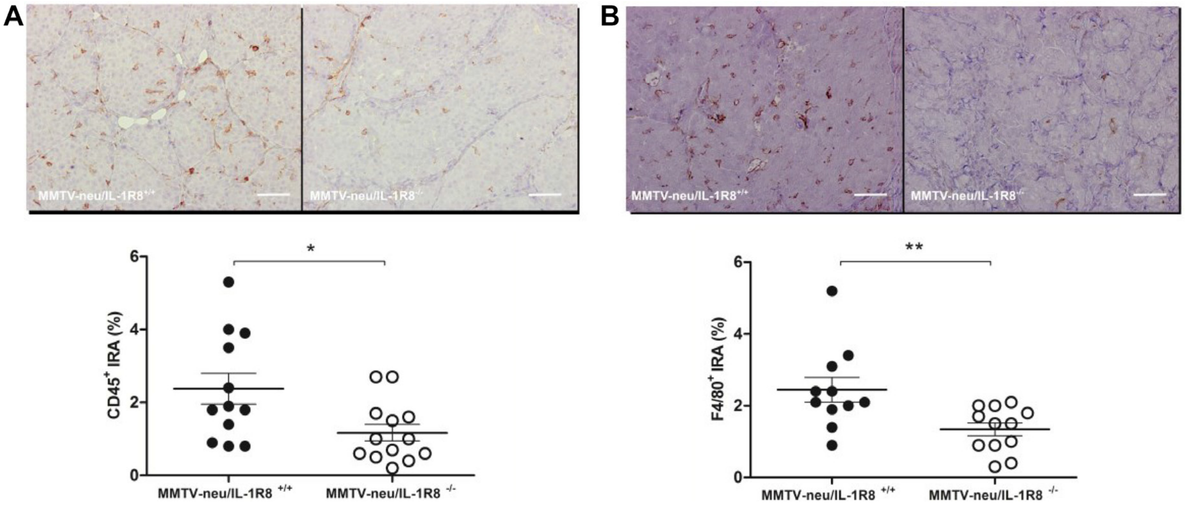

Supplementary Figure 3: MMTV-neu/IL-1R8$^{-/-}$ tumors present lower infiltration of CD45^{+} cells and TAMs. IHC analysis of (**A**) leukocytes (CD45^{+}) and (**B**) macrophage (F4/80^{+}) infiltration in MMTV-neu/IL-1R8$^{-/-}$ and MMTV-neu/IL-1R8$^{+/+}$ tumors. Immunoreactive area (IRA) was determined as % of mean positivity per tumor. Scale bar: 50 µM. *$P < 0.05$ and **$P < 0.01$, unpaired Student's *t*-test.

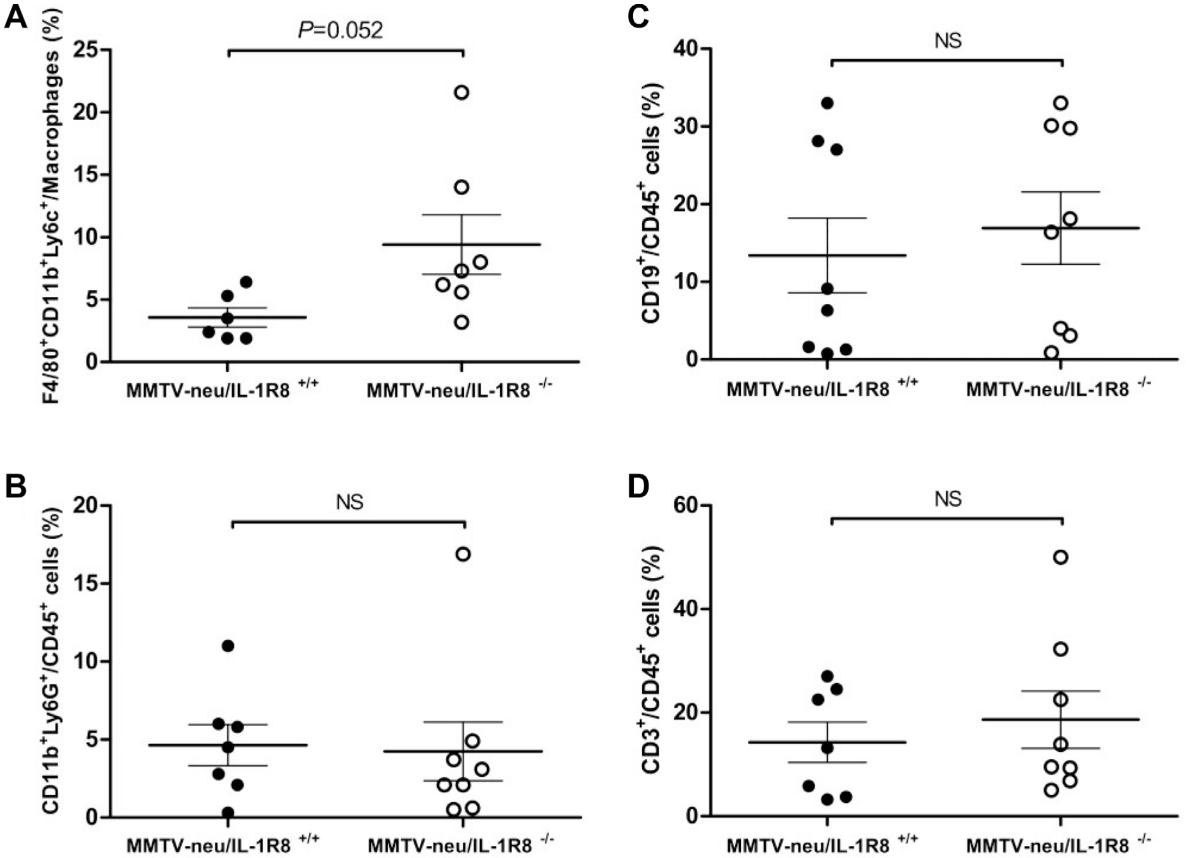

Supplementary Figure 4: Immune infiltrate in MMTV-neu/IL-1R8$^{-/-}$ and MMTV-neu/IL-1R8$^{+/+}$ mammary tumors at 24 weeks of age. (**A**) immature macrophages (F4/80^{+}CD11b^{+}Ly6C^{+}/Macrophages); (**B**) Polymorphonuclear cells (CD11b^{+}Ly6G^{+}); (**C**) B-Cells (CD19^{+}); (**D**) T-Cells (CD3^{+}). (B–D) Results presented as % of CD45^{+} cells. NS = not significant. Statistical analysis: unpaired Student's *t* test.

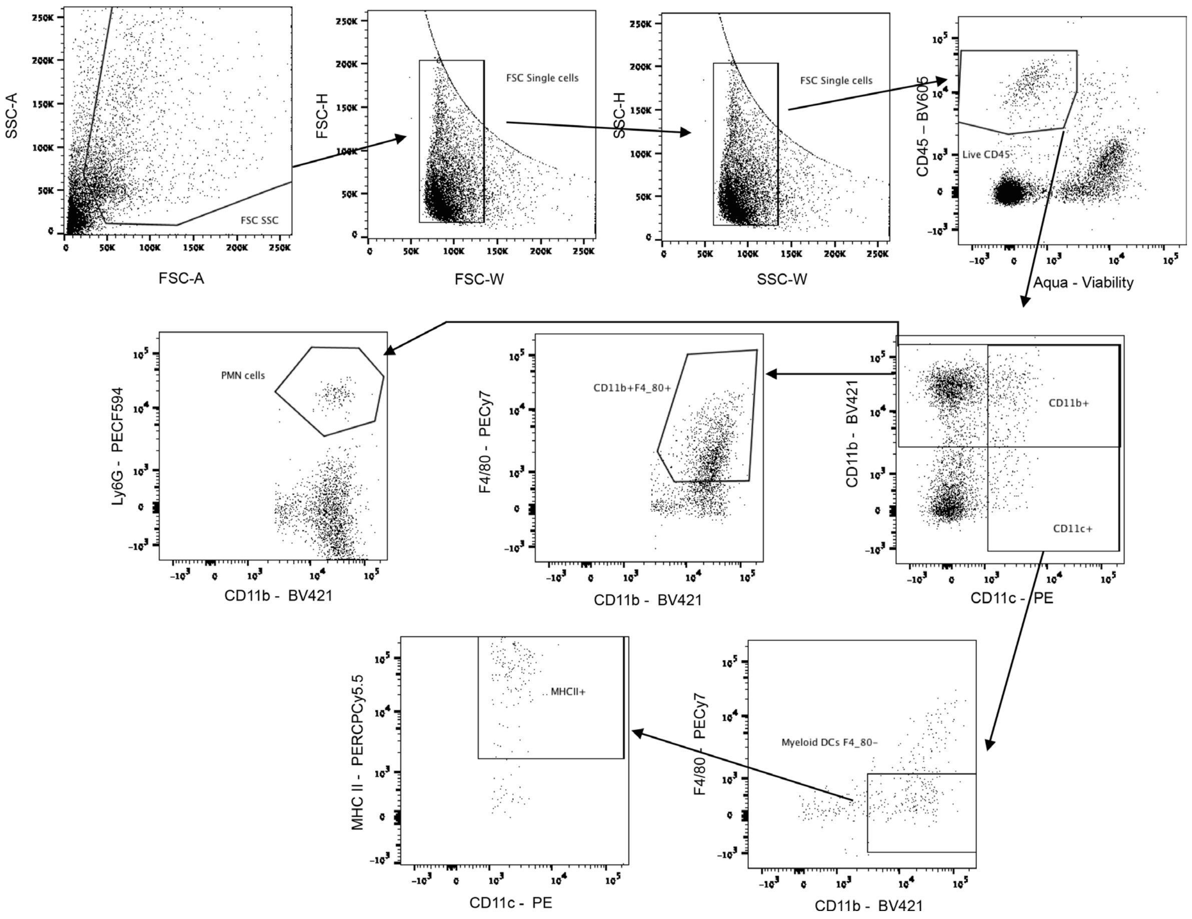

Supplementary Figure 5: Gating strategy for the characterization of myeloid cells.

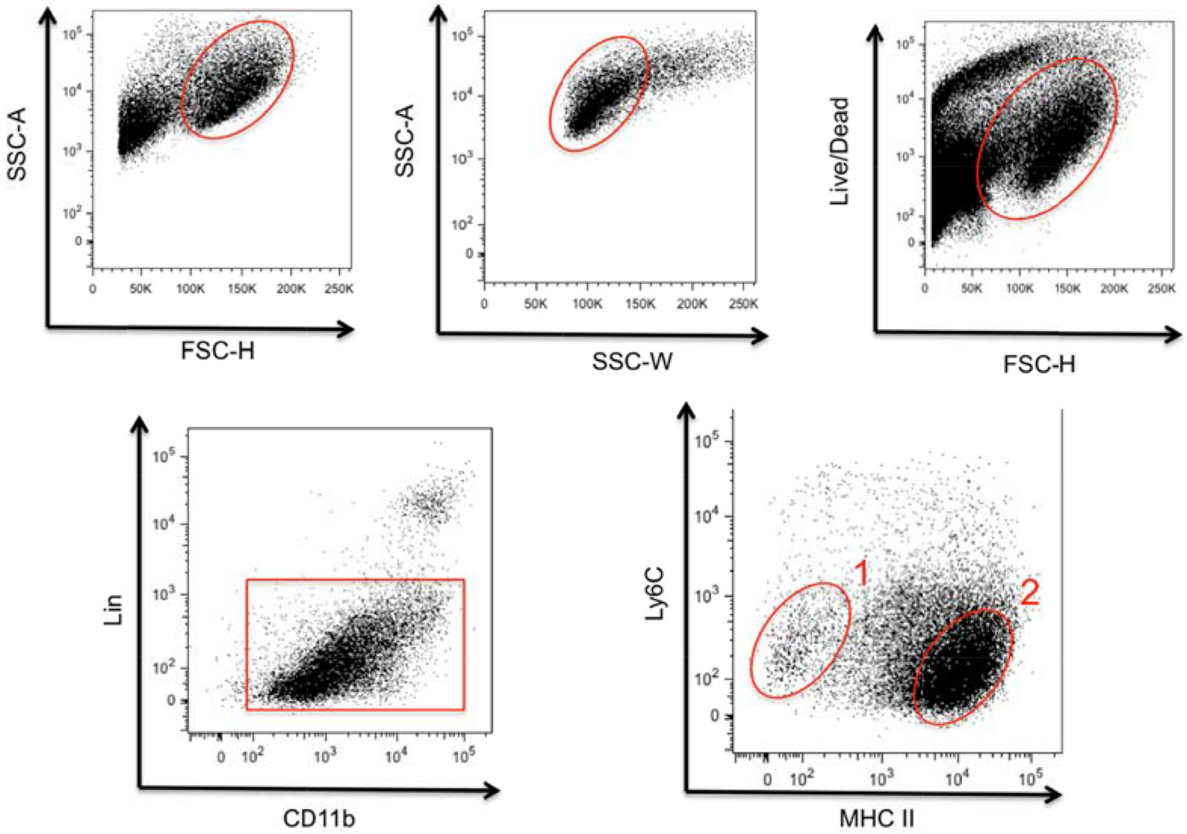

Supplementary Figure 6: Gating strategy to sort TAMs based on Ly6C and MHCII expression. Population 1 (Ly6C^{-}MHCII^{low}) and population 2 (Ly6C^{-}MHCII^{high}), as described by Laoui et al. 2014.

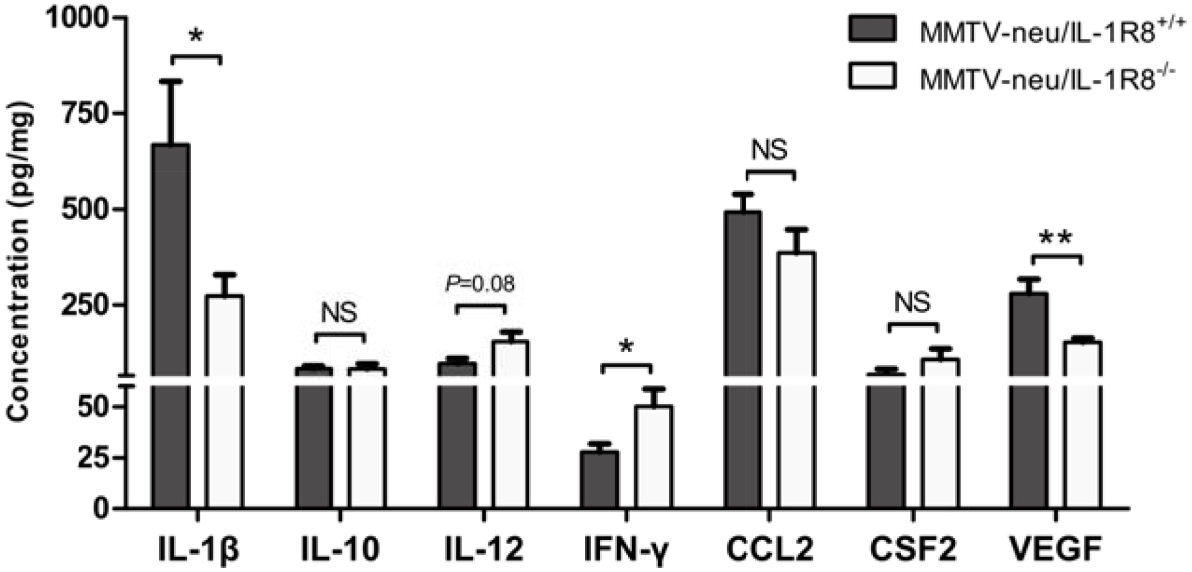

Supplementary Figure 7: MMTV-neu/IL-1R8$^{-/-}$ and MMTV-neu/IL-1R8$^{+/+}$ tumors present distinct levels of inflammatory cytokines. Protein levels of intratumoral cytokines in tumor homogenates from MMTV-neu/IL-1R8$^{+/+}$ and MMTV-neu/IL-1R8$^{-/-}$ mice by ELISA. Statistical analysis: *$P < 0.05$, **$P < 0.01$, unpaired Student's *t* test.

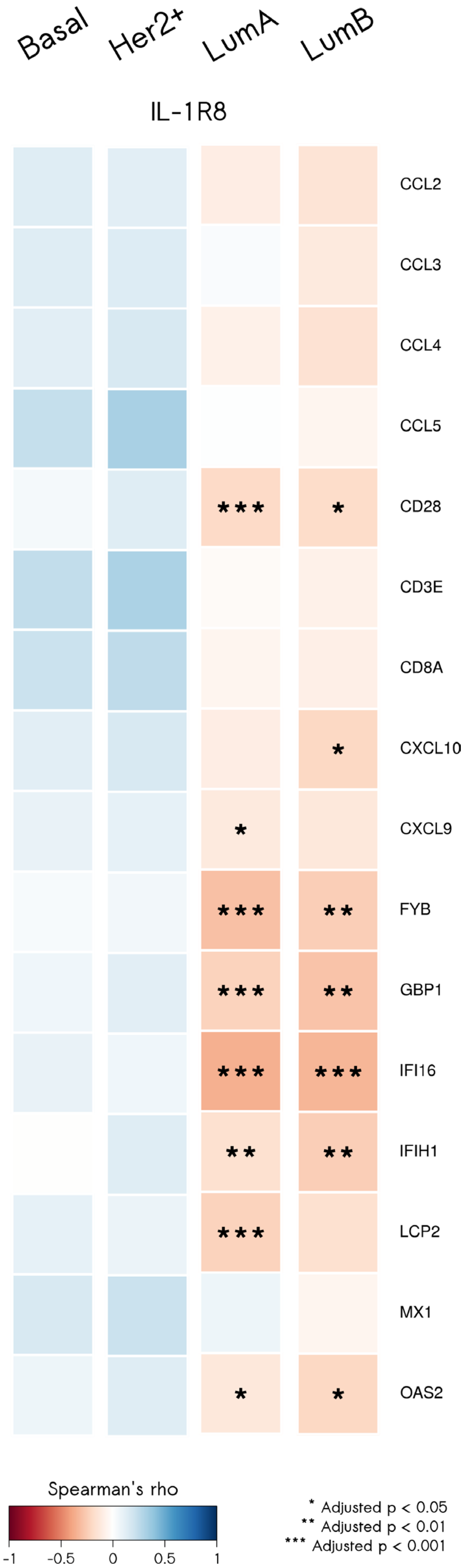

Supplementary Figure 8: Correlation between IL-1R8 and genes from the T-cell Inflamed signature across different molecular breast cancer subtypes. Color scale represents Spearman's rho coefficient and * indicates when the correlation is statistically significant (*adjusted $p < 0.05$, **adjusted $p < 0.01$, ***adjusted $p < 0.001$).
